# Abnormalities in microbiota/butyrate/FFAR3 signaling in aging gut impair brain function

**DOI:** 10.1172/jci.insight.168443

**Published:** 2024-02-08

**Authors:** Sidharth P. Mishra, Shalini Jain, Bo Wang, Shaohua Wang, Brandi C. Miller, Jea Y. Lee, Cesar V. Borlongan, Lin Jiang, Julie Pollak, Subhash Taraphder, Brian T. Layden, Sushil G. Rane, Hariom Yadav

**Affiliations:** 1 USF Center for Microbiome Research,; 2 Department of Neurosurgery and Brain Repair, and; 3 Center for Excellence of Aging and Brain Repair, University of South Florida (USF) Morsani College of Medicine, Tampa, Florida, USA.; 4 Department of Biomedical and Chemical Engineering and Sciences, Florida Institute of Technology, Melbourne, Florida, USA.; 5 Natural Sciences Division, New College of Florida, Sarasota, Florida, USA.; 6 Department of Animal Genetics and Breeding, West Bengal University of Animal & Fishery Sciences, Kolkata, India.; 7 Division of Endocrinology, Diabetes and Metabolism, Department of Medicine, University of Illinois at Chicago, Chicago, Illinois, USA.; 8 Jesse Brown Veterans Affairs Medical Center, Chicago, Illinois, USA.; 9 Diabetes, Endocrinology and Obesity Branch, National Institute of Diabetes and Digestive and Kidney Diseases, NIH, Bethesda, Maryland, USA.; 10 Division of Digestive Diseases and Nutrition, Department of Internal Medicine, USF Morsani College of Medicine, Tampa, Florida, USA.

**Keywords:** Aging, Microbiology, Alzheimer disease, Bioinformatics, G protein&ndash;coupled receptors

## Abstract

Aging-related abnormalities in gut microbiota are associated with cognitive decline, depression, and anxiety, but underlying mechanisms remain unstudied. Here, our study demonstrated that transplanting old gut microbiota to young mice induced inflammation in the gut and brain coupled with cognitive decline, depression, and anxiety. We observed diminished mucin formation and increased gut permeability (“leaky gut”) with a reduction in beneficial metabolites like butyrate because of decline in butyrate-producing bacteria in the aged gut microbiota. This led to suppressed expression of butyrate receptors, free fatty acid receptors 2 and 3 (FFAR2/3). Administering butyrate alleviated inflammation, restored mucin expression and gut barriers, and corrected brain dysfunction. Furthermore, young mice with intestine-specific loss of FFAR2/3 exhibited gut and brain abnormalities akin to those in older mice. Our results demonstrate that reduced butyrate-producing bacteria in aged gut microbiota result in low butyrate levels and reduced FFAR2/3 signaling, leading to suppressed mucin formation that increases gut permeability, inflammation, and brain abnormalities. These findings underscore the significance of butyrate-FFAR2/3 agonism as a potential strategy to mitigate aged gut microbiota–induced detrimental effects on gut and brain health in older adults.

## Abstract 2

<p>Age-related changes in gut microbiota&nbsp;disrupt butyrate/FFAR2/3 signaling linked to&nbsp;impaired&nbsp;intestinal&nbsp;barrier, resulting in&nbsp;leaky gut, inflammation, cognitive decline, depression, and anxiety.</p>

## Graphical abstract

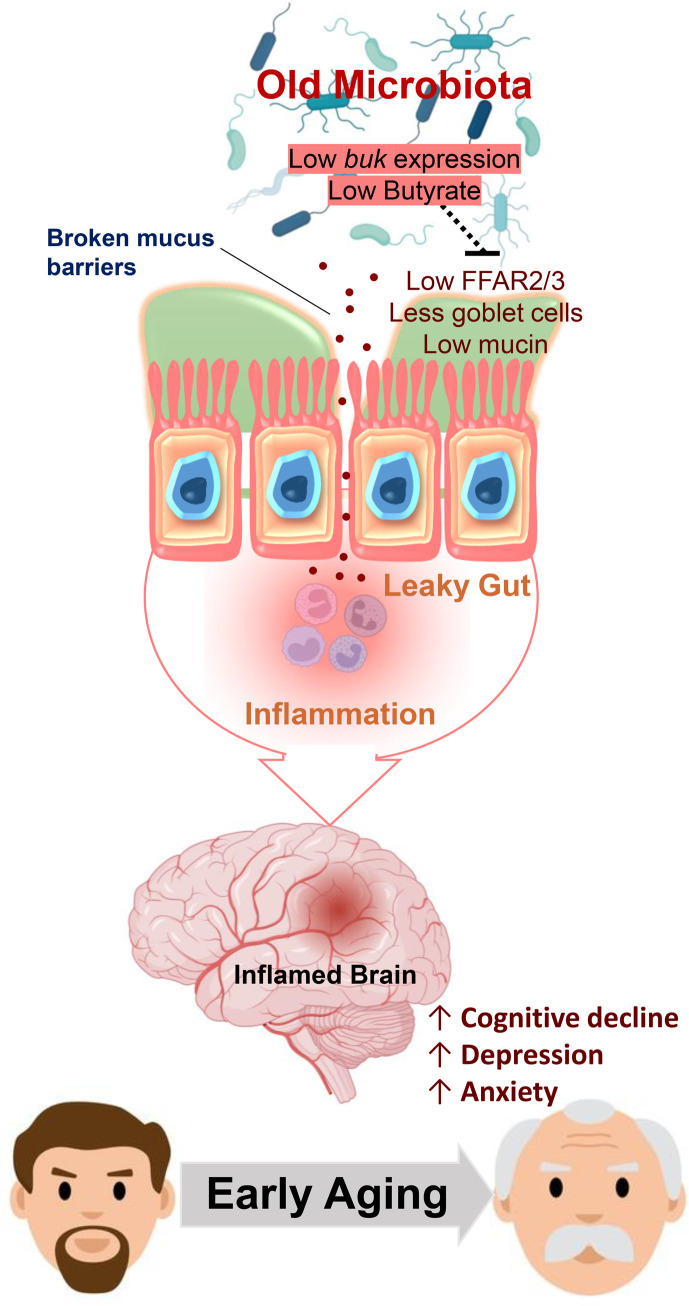

## Introduction

Aging is a key risk factor for prevalent brain abnormalities like cognitive decline, Alzheimer’s disease (AD), depression, and anxiety. We have no effective prevention, or treatment, strategies, imposing a tremendous burden on patients, families, society, and the healthcare system. Among the multitude of putative causes, inflammation is the prime suspect. Biochemical and neuropathologic studies show that inflammatory pathways are activated in the brains of older adults and individuals with AD (1, 2), and clinical studies confirm the correlation between high inflammation and brain dysfunction (3–5). Systemic chronic inflammation begins several years before cognitive decline and dementia are diagnosed (6), but the precise origin and mechanisms are not known.

We and others have shown that high gut permeability or “leaky gut” is a source of inflammation in aging (7, 8). It results from the accelerated breakdown of gut barriers, including fewer tight junction proteins and dampened mucus layer. Increased leakiness allows nonspecific diffusion of microbes, microbial ingredients, metabolites, and antigens from the gut lumen into the blood, promoting the migration of pro-inflammatory molecules and systemic inflammation (9). For example, leakage of lipopolysaccharide (LPS), a cell wall component of Gram-negative bacteria, causes systemic inflammation resulting in endotoxemia (10). Levels of LPS-binding protein (LBP) and soluble CD14 (sCD14), the markers of leaky gut, are markedly higher in older adults and linked to age-associated physical and cognitive decline and increased risk of heart failure (11–13). However, the factors and mechanisms underlying compromised gut barrier functions in the aged gut are not well understood.

The gut microbiome composition is distinct from that in young individuals, often enriched in harmful and depleted of beneficial bacteria (14). A few studies showed that transplanting microbiota from old to young gut instigates leakage, inflammation, and poor health outcomes (15, 16), while another showed opposite phenotypes (17). A more recent study demonstrated that transplanting microbiota from young to old mice reduced leakage and inflammation and ameliorated age-related abnormalities in both gut and brain (18, 19). These contrary results demand further investigation, especially since the mechanisms remain obscure.

Several lines of emerging evidence indicate that microbiota-produced metabolites interact with host cells to mediate both pathologies and benefits in the gut and distant organs, including the brain. However, we do not know *which* metabolites and *how* they contribute to these processes. We also do not know whether, which, and how microbial metabolite signaling contributes to gut and brain abnormalities or whether it can be targeted by dietary or pharmaceutical interventions. We used comprehensive analyses to unravel the causal relationship between microbiota-dependent mechanisms and age-related leaky gut and inflammation to inform translational studies to mitigate adverse outcomes in brain health.

## Results

### Old microbiota exacerbates leaky gut, inflammation, and brain abnormalities, and young microbiota reverses them

#### An older gut harbors a distinct microbiota from that of the young, linked to increased leaky gut and inflammation.

**Figure 1:**
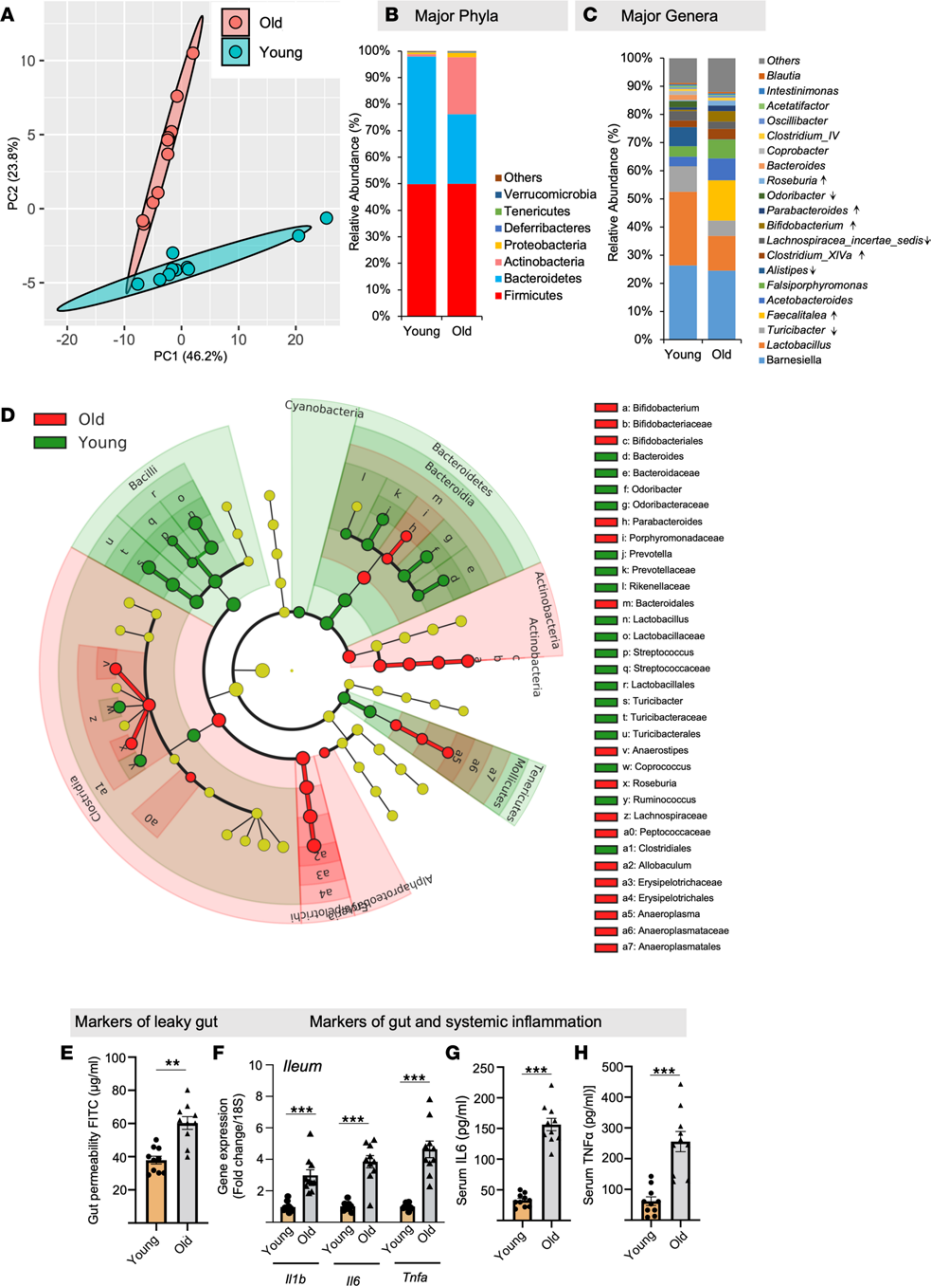
Old mouse gut microbiota is significantly distinct from that of sex-matched controls. (**A**) Principal component analysis (PCA) of β-diversity shows that the microbiota composition significantly differs between old and young feces. Microbiome β-diversity was assessed using the Bray-Curtis dissimilarity index and visualized with PCA. (**B** and **C**) The abundance of major phyla and genera also differs between young and old microbiomes. (**D**) The cladogram of major genera levels in old versus young fecal microbiomes. (**E**–**H**) Old (donor) mice show an increase in gut permeability (FITC-dextran leakage from gut to blood) (**E**); expression of inflammatory genes like *Il1b*, *Il6*, and *Tnfa* (**F**) in the intestine (ileum and colon); and higher circulating IL-6 (**G**) and TNF-α (**H**) compared with young controls. All values represent the mean of 5–10 animals in each group, and error bars represent the standard error of means. Statistical significance was determined using *t* tests (2 tailed) and/or ANOVA (1 or 2 way, as appropriate), and **P < 0.01 and ***P < 0.001 indicate statistical significance.

In agreement with previous studies (20), we demonstrate that the composition of the gut microbiome significantly differs between older mice (≥80 weeks of age) and young mice (≥12 weeks of age) (Figure 1). In brief, the microbiome β-diversity clustering was notably distinct in old and young mice (Figure 1A). Furthermore, the differential phylogenetic abundance of major phyla such as Firmicutes (F) did not show much change, while Bacteroidetes (B) was low, leading to an elevated F/B ratio in the gut of older mice compared with young mice (Figure 1B and Supplemental Figure 1A; supplemental material available online with this article; https://doi.org/10.1172/jci.insight.168443DS1). Similarly, the abundance of microbial genera such as *Barnesiella*, *Lactobacillus*, *Turibacter*, *Alistipes*, *Lachnospira*, and *Odoribacter* decreased in the older gut compared with the young, while *Faecalitalea*, *Clostridium* XIVa, *Bifidobacterium*, *Parabacteroides*, and *Roseburia* increased (Figure 1C). Additionally, linear discrimination analyses (LDA) effect size (LEfSe) using a biomarker discovery tool illustrated that the older microbiota was enriched with *Faecalitalea*, *Acetobacteroides*, *Bifidobacterium*, *Falsimorphimonas*, *Alkaliplus*, *Roseburia*, *Clostridium* XIVa, *Ruminococcus*, and *Parabacteroides* clusters, while the younger microbiota displayed enrichment in *Lactobacillus*, *Alistipes*, *Turibacter*, *Orodoribacter*, *Barnesiella*, *Bacteroides*, *Coprobacter*, *Microbacter*, *Lachnospiracea*, and *Prevotella* with biomarker capacity (Figure 1D and Supplemental Figure 1B). Additionally, the analysis of microbiome characteristics revealed that the microbiota of older mice showed an enrichment in Gram-positive bacteria and a decrease in Gram-negative bacteria (Supplemental Figure 1C). There was also an increase in biofilm-forming bacteria and stress-tolerant microbes when compared with the microbiota of younger mice (Supplemental Figure 1, D and E).

Moreover, the older mice showed a significant increase in gut permeability, as indicated by the elevated diffusion of FITC-dextran from the gut into the bloodstream (Figure 1E). Additionally, signs of inflammation were heightened in the older mice, evident through increased expression of *Il1b*, *Il6*, and *Tnfa* in the intestine (both ileum and colon) (Figure 1F and Supplemental Figure 2), along with elevated IL-6 and TNF-α levels in the serum (Figure 1, G and H). In summation, these findings collectively indicate that the gut microbiota of older mice significantly differs from that of younger mice. These alterations in the microbiota composition are associated with heightened gut permeability and inflammation. However, the question of whether the old microbiota directly causes leaky gut still remains unanswered.

#### Transplanting old microbiota transfers aged phenotypes of microbiota, leaky gut, inflammation, and brain anomalies in young recipient mice, and transplanting young microbiota reverses them.

**Figure 2:**
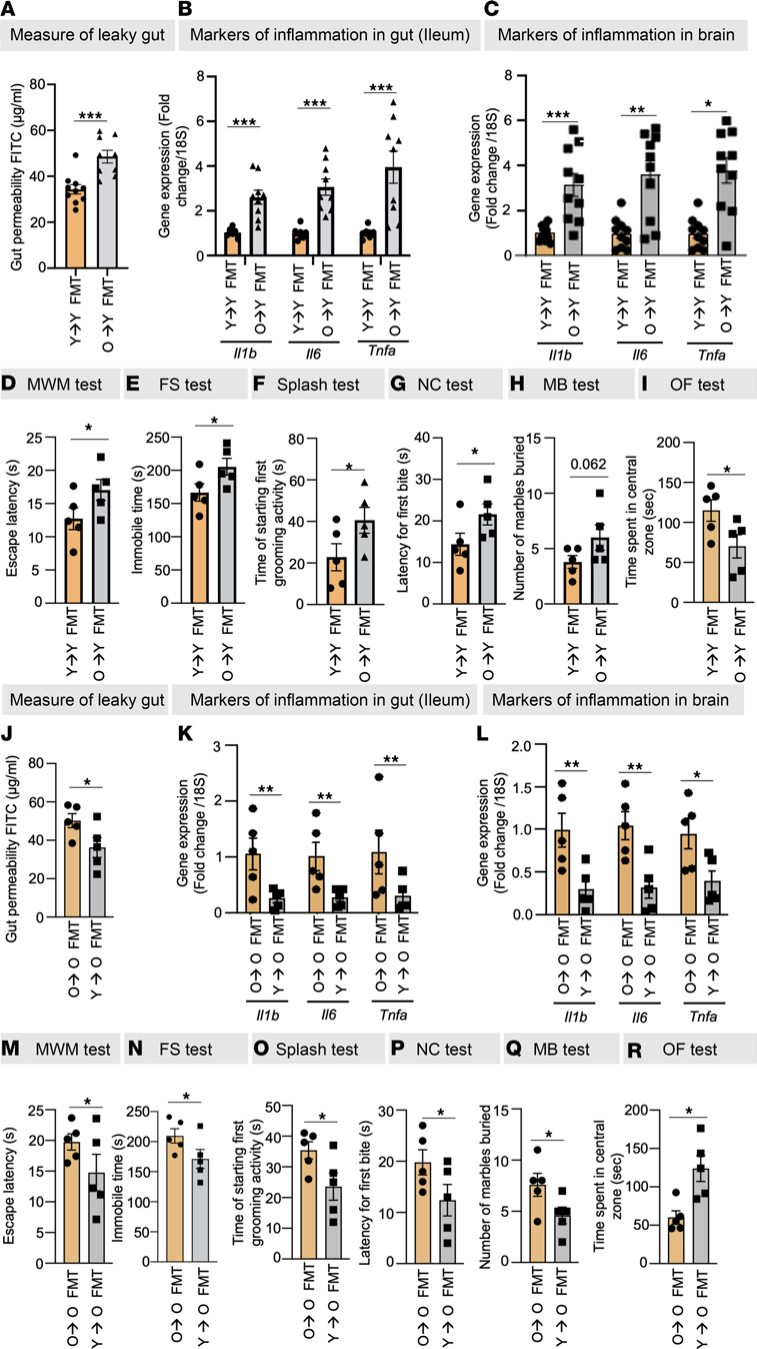
Old gut microbiota transplantation induces age-related leaky gut, inflammation, and behavioral abnormalities, and young microbiota transplantation reduces them. (**A**–**C**) Old FMT significantly increased leaky gut (FITC-dextran leakage) (**A**) and inflammatory markers (*Il1b*, *Il6*, and *Tnfa*) in the ileum (**B**) and brain (**C**) of recipient mice. (**D**–**I**) Old FMT instigated behavioral changes, such as cognitive dysfunction (Morris water maze [MWM] test) (**D**), depression (forced swim [FS] and splash tests) (**E** and **F**), and anxiety (novel cage [NC], marble burying [MB], and open field [OF] tests) (**G**–**I**). (**J**–**R**) Interestingly, young FMT to old mice significantly ameliorated leaky gut (**J**), inflammation in the ileum (**K**) and brain (**L**), and cognitive decline (**M**), depression (**N** and **O**), and anxiety (**P**–**R**) behaviors. All values represent the mean of 5–10 animals in each group, and error bars represent the standard error of the means. Statistical significance was determined using a 2-tailed *t* test, as applicable, and *P* values of *P < 0.05, **P < 0.01, and ***P < 0.001 are statistically significant.

To investigate whether the old microbiota directly causes leaky gut, we conducted fecal microbiota transplantation (FMT) using donors of both young age (≥12 weeks) and old age (≥80 weeks) into recipient mice at approximately ≥12 and ≥80 weeks of age (Supplemental Figure 3). Remarkably, this transplantation resulted in the establishment of recipient gut microbiome profiles that closely resembled those of their respective donors, as evidenced by a range of indicators such as similar β-diversity indices, phylogenetic abundance, cladogram structures, LDA scores, and specific traits like the ratio of Gram-positive to Gram-negative bacteria, biofilm-forming, and stress-tolerant bacterial abundances (Supplemental Figure 4, A–I). Moreover, the old FMT recipient mice of age ≥12 weeks exhibited a substantial increase in both leaky gut indicators (highlighted by elevated levels of LBP and sCD14 in plasma, along with the FITC-dextran assay; Figure 2A and Supplemental Figure 5, A and B) and expression of inflammatory genes (*Il1b*, *Il6*, and *Tnfa*) in both ileum and colon, as well as in blood circulation (Figure 2B and Supplemental Figure 5C). Notably, in comparison with recipients of young FMT, those who received old FMT exhibited heightened neuroinflammation in the brain (Figure 2C), along with elevated cognitive dysfunction (Morris water maze [MWM] test), depression (forced swim [FS] and splash test), and anxiety (novel cage [NC], marble burying [MB], and open field [OF] test) (Figure 2, D–I). Interestingly, transplanting young microbiota to older mice significantly decreased leaky gut markers (FITC-dextran leakage from gut to blood); decreased inflammation in gut, brain, as well as systemic circulation; improved cognitive function; and reduced depression and anxiety behaviors in older recipient mice compared with their respective controls (Figure 2, J–R, and Supplemental Figure 5D). These results indicate that the transplantation of old microbiota transfers the aged phenotype of the donors, encompassing microbiota profiles, leaky gut, inflammation, and brain health ailments, into the young recipient mice, while transplanting young microbiota into old gut reverses these age-related anomalies. However, the intricate mechanisms through which the old microbiota initiates age-related leaky gut and inflammation remained largely undiscovered.

### Old microbiota disrupts intestinal barriers, leading to decreased mucin and leaky gut and inflammation

**Figure 3:**
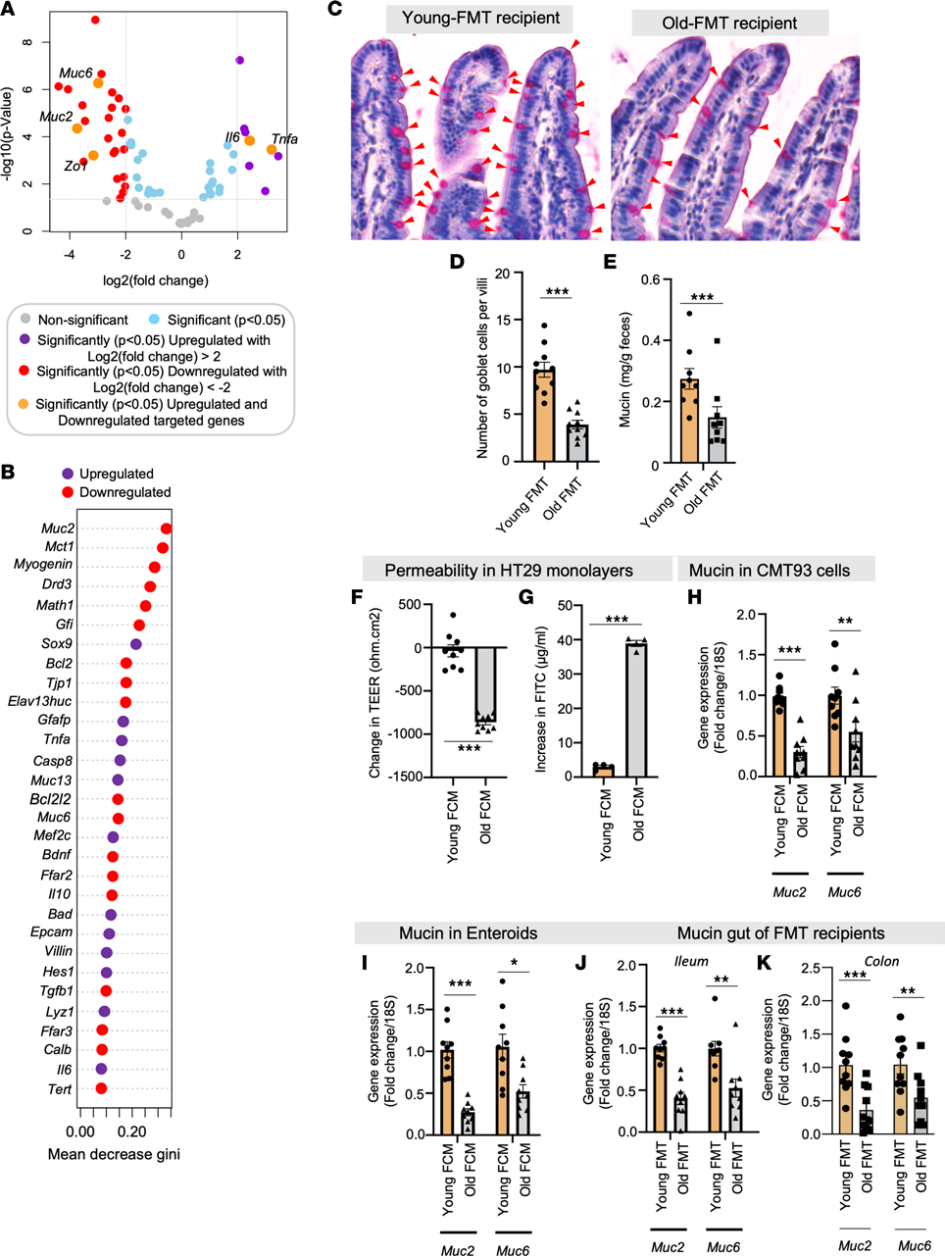
Old microbiota disrupts gut barrier functions by reducing mucin expression and goblet cells in recipient mouse gut. (**A**) Differential abundance data on an intestinal epithelia-related gene array showing significantly reduced expression of mucin genes *Muc2* and *Muc6* and tight junction gene *Zo1* and increased expression of inflammatory genes *Il6* and *Tnfa* in the intestine (ileum) of mice receiving FMT from old mice compared with controls receiving young FMT. (**B**) Random forest analysis shows that *Muc2* was most significantly affected by old FMT. The mean decrease in gini score respresents the importance of the variable in building the model; thus, the higher the value of the mean decrease in gini score, the higher the importance of the variable in the model. (**C** and **D**) Old FMT recipients have significantly fewer goblet cells (periodic acid–Schiff [PAS] staining; red arrows) in their intestinal villi than controls. (**E**) Fecal mucin content was also significantly lower in old FMT recipients than in controls. (**F** and **G**) Treatment with fecal conditioned media (FCM) made from the feces of old mice significantly reduced transepithelial electrical resistance (TEER) (**F**) and increased FITC-dextran permeability (**G**) in the monolayers of human HT29 cells compared with treatment with young FCM. (**H**–**K**) *Muc2* and *Muc6* expression was significantly reduced in goblet-like CMT93 cells (**H**) and enteroids (**I**) treated with old FCM; they resembled the intestines (ileum and colon) of old FMT recipient mice (**J** and **K**). All the values represent the mean of 5–10 animals or 3–4 independent replicates for each group in the cell and enteroid experiments repeated 2–3 times, and error bars represent the standard error of means. Statistical significance was determined using *t* test and/or ANOVA, as applicable, and *P* values *P < 0.05, **P < 0.01, and ***P < 0.001 are statistically significant.

To uncover the mechanisms underpinning the influence of aged microbiota on leaky gut, we performed a gene expression array encompassing 76 genes related to intestinal epithelia (Supplemental Table 1). Our observations unveiled a significant reduction in the expression of essential gut barrier genes, notably mucin 2 (*Muc2*) and Zonulin-1 (*Zo1*), alongside a pronounced upregulation of inflammatory genes like *Il6* and *Tnfa* within the intestines (ileum) of recipients exposed to old FMT, as compared with controls who received young FMT (Figure 3A). Similarly, the expression of these genes also decreased in the intestines of old FMT recipient mice (Supplemental Figure 6A). Further, by using unsupervised random forest analysis, we unveiled *Muc2* expression as the most specifically and significantly diminished in intestines of mice that had received old FMT, in comparison with their counterparts who underwent young FMT (Figure 3B and Supplemental Table 2). This observed shift in gene expression was linked with reduction in the intestinal goblet cells and fecal mucin content among recipients of old FMT, as contrasted with those who received young FMT (Figure 3, C–E, and Supplemental Figure 6, B–E). Importantly, the shifts in gene expression and goblet cell presence in recipient gut directly mirrored the gut of the donor mice (Supplemental Figure 6F).

To elucidate the mechanism and replicate the outcomes of FMT in in vitro and ex vivo settings, we systematically optimized an alternative procedure utilizing human intestinal (HT29) cells and murine small intestinal organoids (enteroids derived from ileum) and treated them with fecal conditioned media (FCM). Similar to the effects observed with old FMTs, the treatment with old FCM significantly induced leaky gut phenotypes in HT29 monolayers. This was evidenced by a notable reduction in transepithelial electrical resistance (TEER) and an increase in FITC-dextran diffusion in the Transwell assays (Figure 3, F and G). Furthermore, the treatment of CMT93 cells (a mucin-producing mouse intestinal cell line) and mouse enteroids with old FCMs resulted in a significant reduction in the expression of gut-specific mucin genes such as *Muc2* and *Muc6* (Figure 3H). This observation closely paralleled the *Muc2* and *Muc6* expression changes observed in the small intestinal organoids (enteroids) and intestines (ileum and colon) of recipients exposed to old FMT (Figure 3, I–K). These findings affirm that the aging microbiota triggers leaky gut by diminishing mucin and goblet cells. Importantly, the FCM reproduces the effects of FMT in cell and enteroid cultures. Consequently, these cultivated systems serve as a credible alternative to in vivo FMTs, allowing for the development of a precise mechanistic comprehension of how the microbiota influences gut barrier functions.

Overall, these results highlight the distinct characteristics of the old gut microbiota, which induce leaky gut with suppression of goblet cells and mucin, thus weakening intestinal barriers. This leads to heightened inflammation in the gut, circulation, and brain, ultimately promoting age-related brain abnormalities in old FMT recipient mice, while transplanting young microbiota to old mice reverses these abnormalities. Nevertheless, the mechanisms involved in the microbiota’s impact on decreasing mucin remained unknown.

### Diminished butyrate levels in the old microbiota due to a reduction in butyrate-producing bacteria lead to a decline in goblet cells and subsequent mucin formation

**Figure 4:**
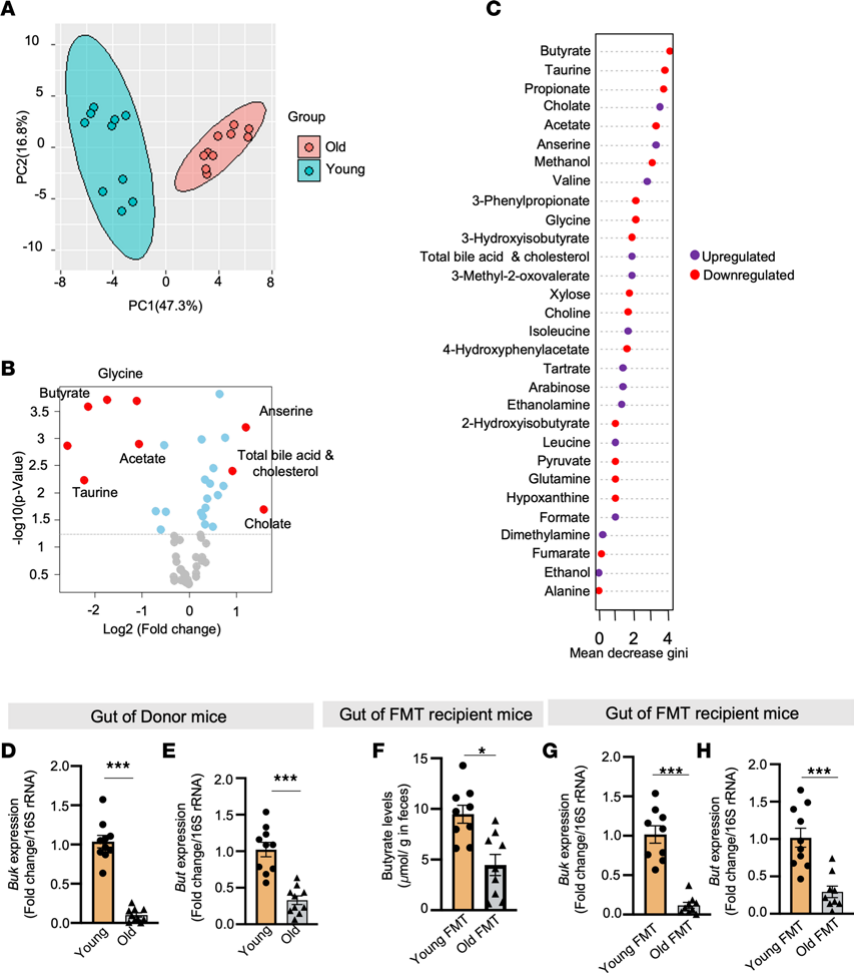
Deficiency of butyrate-producing bacteria lowers butyrate levels in older gut. (**A**) Principal component analysis (PCA) shows a significantly distinct metabolomics signature in the feces of old mice compared with that of sex-, diet-, and genotype-matched young mice. (**B** and **C**) Volcano plot (**B**) and random forest analyses (**C**) of differential abundance show that butyrate was the most significantly reduced metabolite in the old feces. Red dots represent metabolites more than +1 or –1 log_2_ fold-change whereas blue represents statistical significance but log_2_ fold-change is less than –1 or +1. (**D** and **E**) Compared to young feces, old feces showed significantly less expression of the butyrate-producing bacteria abundance markers butyrate kinase (*buk*) (**D**) and butyryl-CoA:acetate CoA transferase (*but*) (**E**) genes. (**F**) FMT transferred the reduced butyrate phenotype from old gut. (**G** and **H**) Reduced expression of *buk* (**G**) and *but* (**H**) was also transferred by old FMT. All the values represent the mean of 5–10 samples in each group. Statistical significance was determined using *t* test, and *P* values *P < 0.05 and ***P < 0.001 are statistically significant.

To further elucidate the mechanism by which the old microbiota suppresses mucin, we focused on microbial metabolites, as these play a crucial role in host-microbiota interactions (21). However, the specific microbial metabolites that undermine mucin barriers and promote leaky gut remain largely unknown. PCA of our metabolomics data revealed a markedly distinctive metabolic signature in the feces of older (~80 weeks of age) donor mice compared with sex-matched young (~12 weeks of age) controls, both fed an identical diet (Figure 4A). Moreover, differential abundance analysis (Figure 4B) and clustering analyses (Supplemental Figure 7) indicated that beneficial metabolites such as short-chain fatty acids (SCFAs) — acetate and butyrate — along with the amino acid taurine, were notably reduced, while substances like anserine, total bile acids, cholesterol, and cholate were significantly elevated in older feces compared with younger counterparts. Subsequent random forest analyses underscored the considerable reduction in butyrate levels (Figure 4C and Supplemental Table 3), followed by acetate (Supplemental Figure 8, A–C). Further, low butyrate levels exhibited the most positive correlation with markers of leaky gut and inflammation, while displaying the most negative association with markers of mucin barrier integrity among all the metabolites (Supplemental Figure 9). Furthermore, among the key SCFAs (acetate, propionate, and butyrate at physiological concentrations of 10 μM, 6 μM, and 6 μM, respectively), butyrate elicited the highest levels of mucin, evident from PAS staining, as well as the expression of *Muc2* and *Muc6* in CMT93 cells and enteroids (Supplemental Figure 10, A–C). Together, these outcomes underscore the predominant influence of butyrate within the older gut milieu; its depletion correlates with increased permeability, inflammation, and compromised mucin levels, albeit without a clear underlying mechanism by which butyrate production decreases in old gut.

Investigating this phenomenon, we initially noted lowered levels of butyrate-producing bacteria and the expression of 2 pivotal butyrate-synthesizing enzymes, butyrate kinase (*buk*) and butyryl-CoA:acetate CoA transferase (*but*), within the older gut (Figure 4, D and E). This suggests a diminished capacity for butyrate production by the microbiome in the aging gut. Notably, reduced butyrate levels and diminished expression of *buk* and *but* genes were also observed in old FMT recipient mice (Figure 4, F–H), indicating that the reduced butyrate phenotype of the aging gut can be transferred to younger recipients. Further, multivariate analyses established a positive correlation among reduced butyrate levels, the expression of *buk* and *but* genes, and *Muc2* expression (Supplemental Figure 11, A–E). Thus, these findings collectively indicate that the decline in butyrate-producing bacteria is directly linked to reduced butyrate production, leading to compromised goblet cells and mucin formations, consequently contributing to heightened leaky gut conditions and inflammation observed in the aging gut.

### Butyrate protects from the leaky gut, inflammation, and behavioral abnormalities caused by the old microbiota, by promoting mucin formation

**Figure 5:**
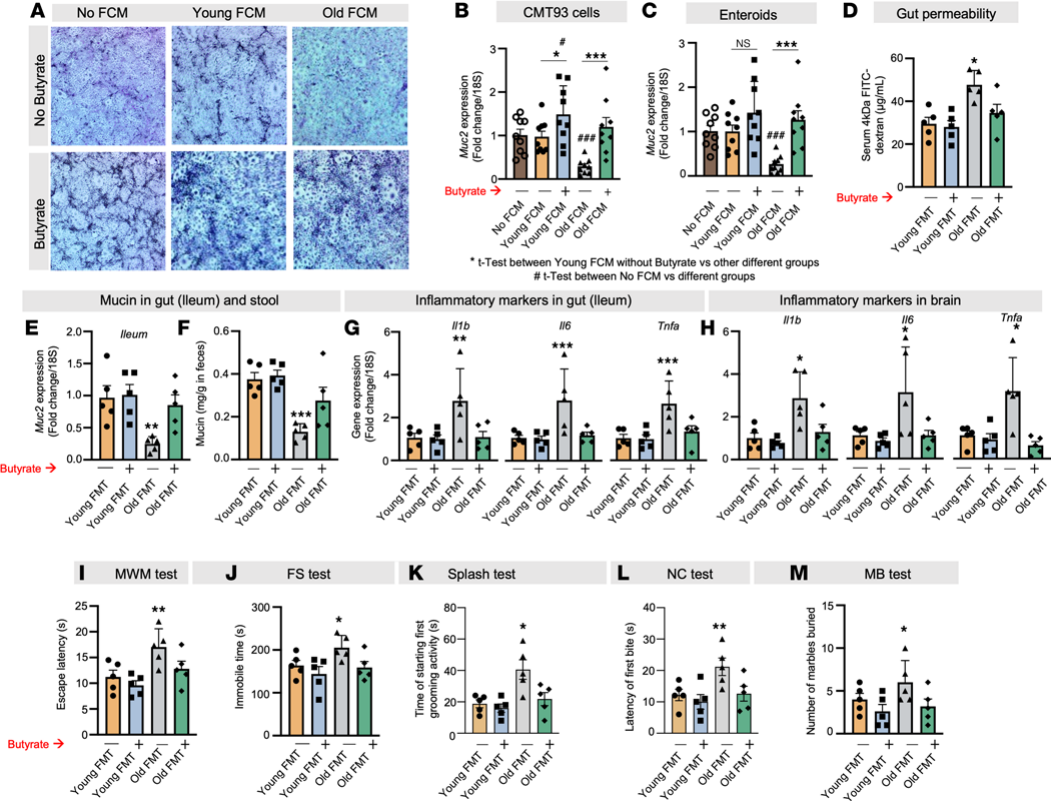
Butyrate treatment promotes gut mucin, protecting against the adverse effects of old microbiota on leaky gut and inflammation. (**A**–**C**) Old FCM reduced mucin accumulation (PAS [blue] staining) (**A**) and *Muc2* expression in both CMT93 cells (**B**) and enteroids (**C**), and butyrate treatment (6 mM) attenuated the decrease. The original magnification of these images was 4×. (**D**–**F**) Butyrate (2%) feeding in drinking water significantly reduced leaky gut (FITC-dextran leakage from gut to blood) (**D**) and increased *Muc2* expression in the ileum (**E**) and fecal mucin content (**F**) of mice receiving old FMT (green) compared with controls not treated with butyrate (grey). Their gut recovered to the point that it resembled that of the young FMT recipient controls (blue). (**G**–**M**) Similarly, old FMT-induced inflammation (*Il1b*, *Il6*, and *Tnfa*) in ileum (**G**) and brain (**H**) along with behavioral abnormalities, such as cognitive dysfunction (**I**), depression (**J** and **K**), and anxiety (**L** and **M**), were significantly reduced in butyrate-treated old FMT recipient mice (green) compared with controls (gray), until their condition resembled that of young FMT recipients (blue). All the values represent the mean of 5–8 animals or 3–4 independent replicates for each group in the cell and enteroid experiments, repeated 2–3 times, and error bars represent the standard error of means. Statistical significance was determined using 2-tailed Student’s *t* test or 1- or 2-way ANOVA, as applicable, and *P* values *^,#^*P* < 0.05, **P < 0.01, and ***^,###^*P* < 0.001 are statistically significant.

It was demonstrated that the application of old FCM significantly diminished mucin in goblet-like CMT93 cells, as indicated by PAS staining (Figure 5A and Supplemental Figure 12), when compared with young FCM treatment. However, treatment with butyrate (6 μM) elevated mucin levels in all three, no-FCM control as well as young and old FCM-treated cells (Figure 5A). Similarly, reduced *Muc2* expression was evident in both CMT93 cells and enteroids treated with old FCM, with butyrate intervention reversing this effect (Figure 5, B and C). Butyrate exhibited superior protection against the reduction in *Muc2* expression in old FCM-treated CMT93 cells in comparison with acetate (Supplemental Figure 13). This demonstrates that butyrate confers protection to goblet cells against the adverse impacts of the old microbiota, thus fostering mucin formation.

To emphasize the physiological importance of butyrate therapy, we demonstrate that administering butyrate through drinking water (at a concentration of 2%) to mice that underwent old FMT significantly reduced gut permeability (Figure 5D and Supplemental Figure 14). It also increased *Muc2* expression in both the ileum and colon and enhanced mucin content in feces, bringing these levels back to those seen in young FMT recipient controls (Figure 5, E and F, and Supplemental Figure 15). Moreover, butyrate intervention significantly suppressed the expression of inflammatory genes (*Il1b*, *Il6*, and *Tnfa*) in both gut and brain and ameliorated cognitive function (MWM test), depressive behavior (FS and splash tests), and anxiety (NC, and MB tests) in mice subjected to old FMT and effectively restored to their baseline states (Figure 5, G–M). In summary, these results highlight that butyrate plays a protective role against the reduction of mucin caused by the aged microbiota. This protective effect effectively mitigates the consequences of age-related microbiota-induced leaky gut and inflammation in both the gastrointestinal and neural systems, ultimately supporting enhanced brain health. However, the exact mechanisms underlying butyrate’s impact on mucin expression required further investigation.

### The old microbiota suppresses FFAR2 and FFAR3, which mediate the effects of butyrate in promoting mucin

**Figure 6:**
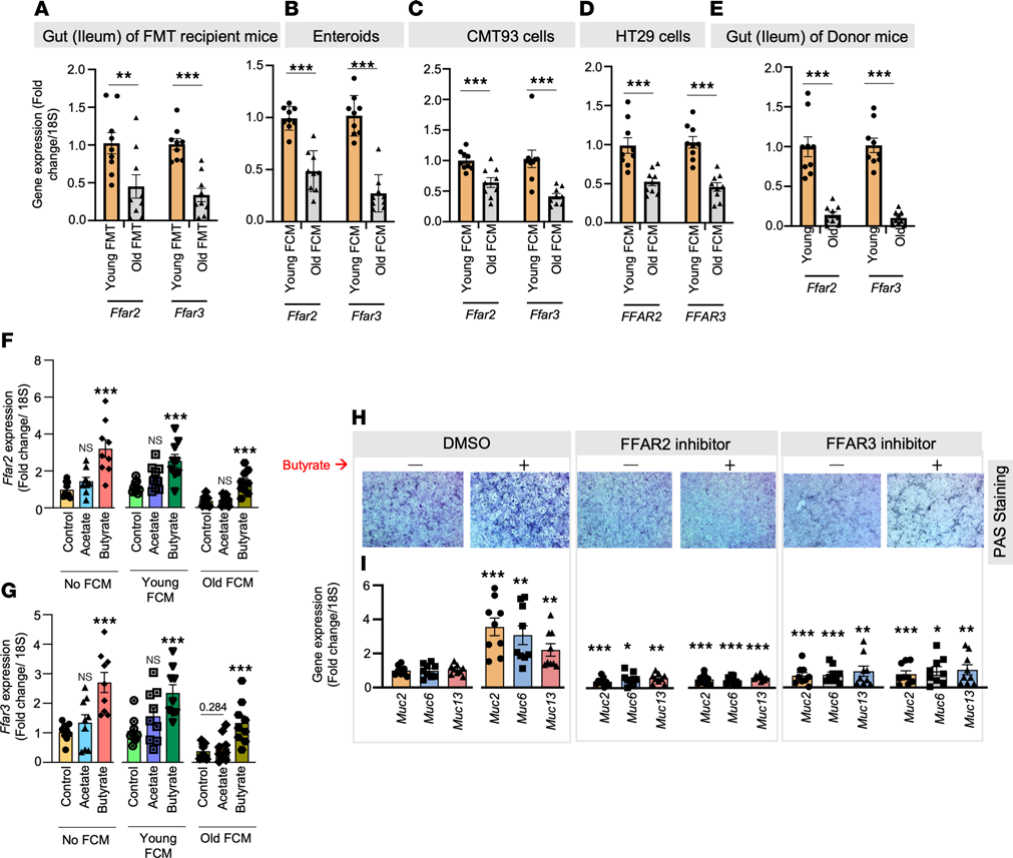
Old microbiota transplantation reduces FFAR2/3 signaling, which attenuates butyrate’s beneficial effect on gut mucin. (**A**) Mice that received old FMT expressed significantly fewer *Ffar2* and *Ffar3* genes than those that received young FMT. (**B**–**D**) *Ffar2/3* expression was also significantly reduced in enteroids (**B**), CMT93 cells (**C**), and HT29 cells (**D**) treated with old FCM compared with controls treated with young FCM. (**E**) Changes recapitulated the suppressed *Ffar2/3* expression in the intestine (ileum) of old donors compared with young controls (orange). (**F** and **G**) Butyrate (6 μM) significantly increases *Ffar2/3* expression in non-FCM and young FCM-treated CMT93 cells and protects from decline in *Ffar2/3* expression old FCM-treated cells, while such changes are not seen in acetate-treated groups. (**H** and **I**) Inhibiting FFAR2 (using CATPB) and FFAR3 (using siRNA) dampened the positive effects of butyrate treatment on mucin accumulation (PAS staining) (**H**) and *Muc2*, *Muc6*, and *Muc13* expression (**I**) in CMT93 cells, indicating that FFAR2/3 signaling mediates butyrate’s increasing mucin. The original magnification for these images was 4×. All the values represent the mean of 5–10 animals or 3–4 independent replicates from each group in cells and enteroid experiments, repeated 2–3 times, and error bars represent the standard error of means. Statistical significance was determined using *t* test and/or ANOVA, as applicable, and *P* values *P < 0.05, **P < 0.01, and ***P < 0.001 are statistically significant.

SCFAs, such as butyrate, activate G protein–coupled receptors like free fatty acid receptor 2 (FFAR2; GPR43) and FFAR3 (GPR41) in host cells (22). However, the role of SCFAs and old microbiota in regulating the expression of FFAR2/3 and their function in maintaining mucin homeostasis is not well understood. Here, we showed that the expression of *Ffar2/3* was significantly diminished in the ileum of old FMT recipient mice, as well as in enteroids, CMT93 (mouse) cells (Figure 6, A–C); correspondingly *FFAR2/3* expression declined in HT29 (human) cells treated with old FCM in comparison with young FMT/FCM-treated controls (Figure 6D). Similarly, *Ffar2/3* expression was notably low in the intestines of old donor mice (Figure 6E). Interestingly, our results indicate that butyrate substantially enhanced the expression of *Ffar2/3* in CMT93 cells treated with old FCM, unlike acetate, which showed no such effects (Figure 6, F and G). This suggests that the deficiency of butyrate in the aged gut could be responsible for the decline in *Ffar2/3* expression. Furthermore, butyrate treatment restored *Ffar2/3* expression. Additionally, inhibiting FFAR2 (using CATPB; 1 μM) and FFAR3 (siRNA) diminished butyrate-induced mucin formation (PAS staining) and the expression of *Muc2*, *Muc6*, and *Muc13* in CMT93 cells and enteroids compared with controls (Figure 6, H and I, and Supplemental Figure 16). In conclusion, our findings demonstrate that the old microbiota suppresses *Ffar2/3* expression, which is linked with reduced mucin formation, leading to leaky gut and inflammation. On the other hand, butyrate increases the expression of *Ffar2/3*, thereby mediating its role in stimulating mucin.

### Deficiency of Ffar2 and Ffar3 in the gut exacerbates early aging–like changes in both the gut and brain

**Figure 7:**
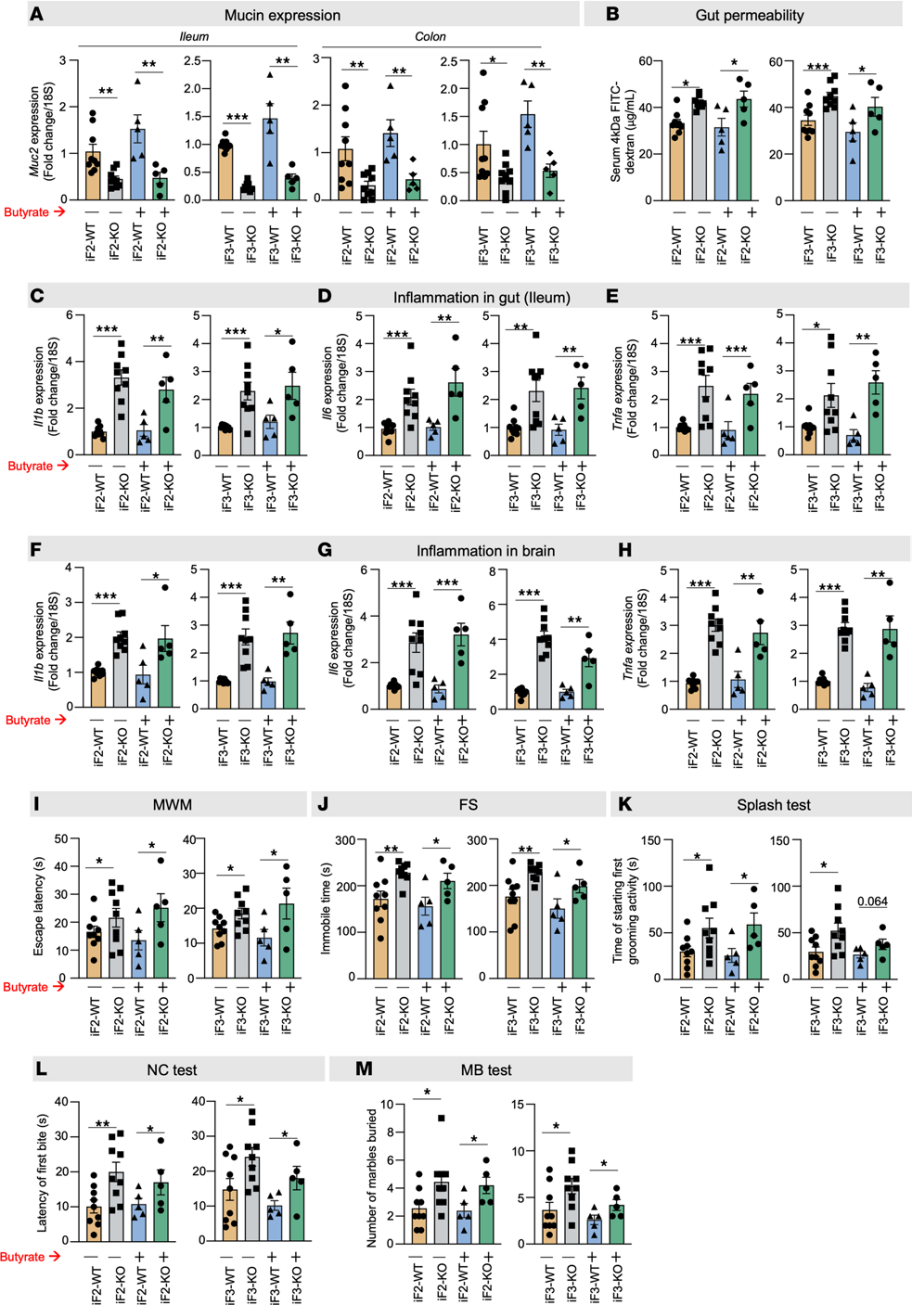
FFAR2/3 deficiency in the gut exacerbates early aging in the brain, including neuroinflammation and behavioral changes. (**A**) The expression of *Muc2* in both ileum and colon was significantly lower in the 7-month-old, intestine-specific FFAR2 (iF2) and FFAR3 (iF3) knockout (KO) mice compared with their age- and sex-matched wild-type (WT) controls. (**B**–**E**) In line, the gut permeability (FITC-dextran leakage) (**B**) was higher and the expression of inflammatory genes (*Il1b*, *Il6*, and *Tnfa*) (**C**–**E**) was lower in the intestine of the 7-month-old iF2/3 KO mice than in WT controls. (**F**–**M**) The brains of the 7-month-old iF2/3-KO mice showed significantly higher levels of inflammatory markers (*Il1b*, *Il6*, and *Tnfa*) (**F**–**H**) along with cognitive decline (MWM test) (**I**), depression (FS and splash tests) (**J** and **K**), and anxiety-like behaviors (NC and MB tests) (**L** and **M**) compared with WT controls. (**A**–**M**) No significant changes in *Muc2* expression (**A**), gut permeability (**B**), inflammation in the gut (**C**–**E**) and brain (**F**–**H**), and behavioral changes (**I**–**M**) were seen in iF2/3-KO mice compared to WT controls. All the values represent the mean of 5–10 animals in each group, and error bars represent the standard error of means. Statistical significance was determined using ANOVA and/or *t* test, and *P* values *P < 0.05, **P < 0.01, and ***P < 0.001 are statistically significant.

Mice lacking *Ffar2* (*Ffar2*^fl/fl^ Vil^Cre+^; iF2 KO) and *Ffar3* (*Ffar3*^fl/fl^ Vil^Cre+^; iF3 KO) in their intestines, driven by *Villin*-Cre, exhibited reduced mucin expression (*Muc2* gene) and increased gut permeability (FITC-dextran diffusion) at 7 months of age compared with age- and sex-matched wild-type (WT) littermates (Figure 7, A and B, and Supplemental Figure 17). Additionally, iF2-KO and iF3-KO mice displayed significantly higher levels of inflammatory markers (*Il1b*, *Il6*, and *Tnfa*) in their gut (both ileum and colon) and brains compared with WT controls (Figure 7, C–H, and Supplemental Figure 18, A–C). Notably, both KO mouse strains also showed significantly elevated behavioral abnormalities, including cognitive dysfunction (MWM test), depression (FS and splash tests), and anxiety (NC tests and MB test) (Figure 7, I–M). Importantly, the beneficial effects of butyrate in mitigating leaky gut, enhancing mucin expression, reducing gut and brain inflammation, and ameliorating behavioral abnormalities were attenuated in both iF2- and iF3-KO mice (Figure 7, A–M). These findings collectively highlight that compromised FFAR2/3 signaling in the gut contributes to impaired mucin formation, resulting in leaky gut, gut and brain inflammation, and the development of age-related behavioral abnormalities, ultimately promoting an early-aging phenotype.

## Discussion

Chronic low-grade inflammation is a hallmark of aging biology, a key risk factor for aging-related disorders, and, as the Geroscience hypothesis proposes, a prominent target for reducing them (3). However, its etiology with aging remains ill-defined. Aged mice and humans display an increased gut permeability (“leaky gut”) linked to increased inflammation and poor health (4). Emerging evidence including our data indicate that the old gut harbors an abnormal microbiome (23–25). We and others have demonstrated that (a) abnormal microbiota from old gut causes the leaky gut and inflammation that are linked to impaired brain health (16), and (b) young FMTs reverse these age-related abnormalities (15, 18, 26). However, others showed that old FMTs in germ-free mice had beneficial effects, including increased neurogenesis (17). These contrary results must be explained, and for that, we must precisely define the relevant mechanisms.

Here, we show that the old microbiota suppresses mucin formation, heightening leaky gut and related inflammation and brain health anomalies. Further, our metabolomics analyses revealed that old microbiota produces less butyrate because of diminished butyrate-producing bacteria in older gut. Replenishing butyrate mitigates leaky gut, and related inflammation, as well as behavioral dysfunctions. We discovered that old microbiota suppresses FFAR2/3 signaling, which corresponds to downregulation of mucin formation, resulting in leaky gut. Inducing deficient FFAR2/3 signaling in young mouse gut also instigates a leaky gut, inflammation (in both gut and brain), and brain dysfunction while attenuating the beneficial effects of butyrate therapy. Thus, FFAR2/3 agonism presents a target for mitigating age-related abnormalities of the gut/brain axis.

Leaky gut occurs because of structural disruptions in gut barriers, primarily linked with reduced mucin and tight junction proteins (TJPs) (27). Mucin forms a physical barrier by forming a thick mucus layer, while TJPs adhere to intestinal epithelial cells. Intestinal goblet cells produce mucin as part of a thick, viscous mucus layer that facilitates the selective diffusion of small molecules from the gut lumen to intestinal epithelial cells (28). While it prevents any direct bacterial interaction with intestinal epithelial cells, it allows bacteria to colonize on its surface (29). Mucin also serves as a food source for numerous gut bacteria, such as the mucin-degrading bacteria *Akkermansia* (30). Conversely, mucin depletion permits microbe interaction with intestinal epithelial cells, which initiates TJPs’ degradation to increase leaky gut and inflammation (31). *Muc2* is the major isoform in the intestine along with *Muc6* and *Muc13* (32). *Muc2*-deficient mice develop severe colitis and gut inflammation (33). In aging rodent and primate models, a thin mucin lining links the resulting gut inflammation to age-associated diseases (34–36). Taken together, maintaining a healthy mucus layer is critical for ideal gut barrier function with advancing age. Our FMT studies reveal that the old gut microbiota reduces expression of mucin and TJPs, but whether one is the direct effect and the other a consequence remains unknown. Our intestine-specific gene array, including a wide range of mucin and TJP genes, in mouse intestine, small intestinal organoids, and human (HT29) and mouse (CMT93) intestinal cells, revealed that *Muc2* expression was predominantly and most remarkably affected, suggesting that older microbiota diminished mucin formation, which corresponded to induced leaky gut and inflammation. Additionally, we also showed that FMTs and FCMs of old microbiota reduce both the number of goblet cells and mucin expression in gut epithelia, suggesting that it initiates the disruption of mucin/gut barriers by reducing goblet cells, which decreases mucin. However, the mechanisms and molecular drivers of reduced goblet cells and mucin levels in the aging gut remain poorly understood and need further comprehensive studies to be conducted.

Our metabolomics analyses reveal that beneficial metabolites, such as SCFAs, predominantly butyrate, are dramatically depleted in the gut (feces) of older mice, similar to others (37). Although butyrate is the most studied microbiota metabolite for its myriad health effects (38–40), its effects and mechanisms in aging are not well known. Its production and contributions to old versus young FMT studies have been questioned (17). Butyrate levels are low in many human disease states (41, 42), often linked to reduced fiber intake. However, our mice were fed identical diets, so the low butyrate levels in the gut of old mice cannot be attributed to food and its intake. Instead, we show its connection to the suppression of butyrate-producing bacteria such as *Barnesiella*, *Odoribacter*, *Lactobacillus*, *Turibacter*, *Alistipes*, and *Lachnospira* (43–47). We also demonstrate that restoring high levels of butyrate boosts the gut’s resilience against old microbiota-induced leaky gut and inflammation and promotes healthy aging behaviors.

Butyrate acts on host cells by several known mechanisms, such as (a) entering the cells through monocarboxylate transporters 1/2, which changes intracellular metabolic flux; (b) inhibiting histone deacetylases (HDACs); or (c) activating membrane receptors (FFAR2/3 signaling) (22). How it regulates mucin barrier functions remains unknown. We show that expression of the *Ffar2* and *Ffar3* genes is low in older gut, and that deficit is transferred via FMTs or FCM from old gut. Interestingly, our findings revealed that butyrate deficiency is the primary cause of *Ffar2/3* expression decrease in older gut, while acetate (another beneficial microbial metabolite) does not impact *Ffar2/3* expression; however, it induces mucin similar to butyrate. This may be because butyrate pronouncedly contributes in *Ffar2/3* expression, while both acetate and butyrate can stimulate FFAR2/3 receptor when they are expressed on goblet cell surface to stimulate mucin formation. As butyrate is one potent HDAC inhibitor (48), and the regulation of *Ffar2/3* expression may be through butyrate-mediated HDAC inhibition, this mechanism needs further studies. Inhibiting FFAR2/3 signaling dampened butyrate’s beneficial effects on mucin expression, further supporting that FFAR2/3 signaling is responsible for maintaining and stimulating mucin expression. Mucin expression is significantly low in intestine-specific FFAR2- and FFAR3-KO mice, which show elevated leaky gut and inflammation at a relatively young adult age (7 months old), and butyrate therapy had no beneficial effects in these KO mice. These findings suggest that compromised FFAR2/3 signaling recapitulates phenotypes typically seen in the old gut and mediates butyrate actions, underscoring its potential importance in the early-aging gut phenotype and as a therapeutic target to mitigate age-related gut and brain dysfunctions.

Brain health deteriorates with aging, and cognitive decline, depression, and anxiety are prevalent among elderly individuals (49). The factors that increase the risk of these debilitating abnormalities remain largely unknown. Increased systemic inflammation is a prime suspect because it is detected several years before cognitive decline and other behavioral changes and is found in the brains of older adults with cognitive decline, dementia, depression, and anxiety (50), but its precise source is not known. We study the abnormal microbiota of older gut as a detrimental source of inflammation locally and in the systemic circulation. Transplantation of old microbiota promotes neuroinflammation and behavioral abnormalities in recipient mice by promoting leaky gut. This occurs because of the diminished capacity of the old microbiota to synthesize butyrate, linked to a suppression of FFAR2/3 expression, which is associated with reduced mucin expression and weakening of gut barriers. We also show that FFAR2 and FFAR3 deficiency in the gut exacerbates brain inflammation, poor cognitive function, and depressive and anxious behaviors in relatively young (7-month-old) mice, suggesting that microbiota-induced abnormalities in the gut accelerate age-related behavioral abnormalities.

Our pioneering and comprehensive analyses establish the mechanisms by which old microbiota exacerbates aging-related abnormalities in the gut and brain. However, some limitations open the door for future studies. For example, we discovered that old microbiota reduces goblet cells, hence mucin, but how is still not clear. We do not know why butyrate and butyrate-producing bacteria are depleted in the older gut, even when young and old host eat an identical diet. Despite the transfer of deficient butyrate-producing function from old to young mice, we do not know exactly which microbial cells are responsible. We selected butyrate to pursue our mechanistic studies because (a) the degree of decrease in butyrate levels is highest in both old donors and old FMT recipients; (b) it showed highest negative correlation with markers of leaky gut and inflammation and the highest positive correlation with mucin expression among all the metabolites; and (c) it stimulated the highest mucin levels and expression of mucin genes compared with acetate and propionate in gut cells. However, whether and how butyrate directly affects brain cells (neurons and microglia) require further study. In addition, it was also not noted that changes in behavioral studies may be related to motor function, in addition to neuronal functions. This should be further investigated to determine whether and how microbiota impact physical function compared with cognitive function, which are two of the most debilitating health problems in older adults.

Overall, we demonstrate that in the old gut microbiota, reduced goblet cells and mucin expression deplete gut barriers to promote leaky gut and inflammation. Old microbiota contains low butyrate-producing bacteria, resulting in low butyrate production in gut, and suppresses FFAR2/3 signaling, which in turn weakens mucin and gut barriers. The deficiency in butyrate-FFAR2/3 signaling in the gut develops early-aging phenotypes in both the gut and brain. Butyrate therapy rescues the old microbiota-induced phenotypes by activating FFAR2/3 signaling, suggesting that butyrate supplementation and/or FFAR2/3 agonism represent a potential approach for reversing leaky gut, inflammation, and behavioral abnormalities that plague older adults.

## Methods

### Chemicals, reagents, and instruments

Supplemental Table 4 provides specific information about the chemicals, reagents, and instruments used, including catalog numbers and vendors.

### Donor mice

We purchased 10-week (young donor, *n* = 10) and 78-week (aged donor, *n* = 10) C57BL/6J (B6) male mice from Jackson Laboratory. After acclimatization for 2 weeks in the animal vivarium under a 12-hour light/12-hour dark cycle, fresh fecal samples were collected, snap-frozen, stored in the –80°C freezer, and used for 16S metagenomics, metabolomics, FMT, and FCM studies, as described below. The gut permeability assay was done at the age of ~12 weeks in young and ~80 weeks in old mice, as described below. Mice were euthanized; blood was collected for serum/plasma isolation; and intestine and brain tissues were collected for histology and gene expression studies, as described below.

### FMT

We used mice of 10–12 and 78–82 weeks of age (conventional B6 gut-cleansed mice) instead of germ-free mice in our studies because the latter have several gut abnormalities that could have confounded results. We used our optimized, published protocol for gut-cleansing experiments in aging studies (51–53). To gut-cleanse the recipient mice (10–12 weeks of age), we gave them an antibiotic cocktail (1 g/L ampicillin, 1 g/L metronidazole, 1 g/L neomycin, and 0.5 g/L vancomycin) for 4 days in their drinking water. On the fourth day, all the mice fasted for 4 hours, and 4 doses of polyethylene glycol (PEG) were given at 20-minute intervals using oral gavage. PEG physically cleanses the gut and reduces up to 95% of microbes. After recipient cleansing, we prepared the donor fecal slurry (200 μL) using snap-frozen fecal samples (500 mg) dissolved in 5 mL of reduced PBS (supplemented with 0.1% Resazurin, w/v, and 0.05% l-cysteine-HCl) in an anaerobic chamber (54). We then transplanted young donor microbiota into 9 mice and old donor microbiota into 9 mice. We continued giving the recipients 1 dose of fecal slurry every day for an additional 4 days to stabilize their microbiome and maintain its close resemblance to the donors’. Gut permeability assays, behavioral measurements, blood collection, and tissue analyses followed the FMTs (Figure 1E).

### Butyrate therapy studies

Old and young microbiota were transplanted into 10- to 12-week-old B6 gut-cleansed mice, as described above. After randomization, half of the old FMT recipients (*n* = 5) were administered butyrate (2%) in their drinking water for 2 weeks after the day of gut cleansing, and the other half drank normal water. Recipients of young FMT, receiving no other treatment, were used as controls. The drinking water was changed every other day, and both water and food (normal chow) were fed ad libitum. Gut permeability assays, behavioral measurements, blood collection, and tissue analyses were done after 2 weeks of FMT and butyrate treatment.

### iF2-KO, iF3-KO, and WT mouse studies

Homozygous *Ffar2*^fl/fl^ and *Ffar3*^fl/fl^ mice (obtained from University of Illinois at Chicago) were bred with Villin (Vil)^Cre+^ heterozygous mice to develop the intestinal tissue-specific iF2-KO (*Ffar2*^fl/fl^ Vil^Cre+^) and iF3-KO (*Ffar3*^fl/fl^ Vil^Cre+^) mice. At 7 months of age, they were compared for mucin barriers, gut permeability, inflammation, and behavioral analyses, with their WT counterparts, iF2 WT (*Ffar2*^fl/fl^ Vil^Cre–^) and iF3 WT (*Ffar3*^fl/fl^ Vil^Cre–^), while fed normal chow and drinking water ad libitum. However, the 7-month-old iF2/3-KO and WT mice were also treated with butyrate (2%) in their drinking water for 2 weeks. Gut permeability assays, behavioral measurements, blood collection, and tissue analyses were done in both untreated and butyrate-treated mice, as described below.

### Gut permeability assay

Mice were prefasted for 4 hours and administered 4 kDa FITC-dextran (MilliporeSigma) at a concentration of 1 g/kg body weight through oral gavage. After an additional 4 hours of fasting, blood serum was collected from the tail vein to measure FITC fluorescence at 485 nm excitation and 530 nm emission wavelength using a 96-well plate. FITC concentration was determined using a standard FITC curve, as described in our earlier publications (8, 52, 53, 55, 56).

### Behavioral analyses in mice

A battery of behavioral tests to determine age-related cognitive decline (MWM test), depression (FS and splash tests), and anxiety (NC and MB tests) were performed by lab staff, blinded to groups, as described below.

#### MWM test.

At the center of a big, deep, circular tub filled with water at a temperature of ~26°C, a platform sat 1 inch above water level. The tub was marked with 4 directions (directions 1, 2, 3, and 4) at equal distances pointing toward the center. Before the actual test, the mice were trained for 3 days. Each mouse was put into the water tail-first at any of the 4 direction points and allowed to swim in search of the platform. The time it took to reach the platform was recorded. If the mouse failed to find the platform within 60 seconds, it was guided to it in approximately 60 seconds. This training was conducted twice from each direction. The mouse was then dried, and the training was repeated with all the other mice. On the day of the test, the water was rendered opaque by adding nonfat dry milk. Each mouse underwent 12 trials (3 trials from each direction), with the first direction selected randomly. We followed the following directional pattern with all the mice: trial A, 1, 2, 3, 4; trial B, 2, 4, 3, 1; and trial C, 3, 2, 1, 4. The time the mouse took to reach the platform in each trial (i.e., escape latency in seconds) was recorded from the captured video, blinded to experimental condition.

#### FS test.

A clean, 5-liter plastic beaker was filled with water to a depth of 13–14 cm (~5–6 inches) at a temperature between 25°C–28°C. A mouse was picked up by the tail and gently dropped in the center. Its performance was videoed for 6 minutes, and then it was transferred to a new cage filled with soft paper towels and dried before it was returned to its original cage. The videos were interpreted, blinded to mouse condition, according to immobile time floating with no movement of forelimbs or hind limbs recorded.

#### Splash test.

Fresh 10% sucrose solution in clean laboratory-grade water was prepared. After the mice had acclimatized in new cages for 1 hour, they were splashed on the snout with 200 μL of the sucrose solution twice and returned to the cage. After 5 minutes, the mouse was returned to the original cage. The time to start of first grooming activity (in seconds) was recorded from the video.

#### NC test.

The mice fasted overnight and the next day were brought to the procedure room, where they were placed individually in new cages set with feeder/wire. A big pellet of high-fat diet was placed on the feeder/wire, and the time a mouse took to bite into it, or the latency of the first bite, was recorded. The data analysis was blinded. For any mice that did not take a bite within 45 minutes, the time to first bite was considered 45 minutes.

#### MB test.

Mice were acclimatized in new cages for 2 hours. Taking care not to scare or disturb the animals, 12 marbles were placed in each cage; they had to be equidistant and in the same order of 3 × 4 along the width × length of the cage. The lid was then closed for 30 minutes. The procedure room was silent and without movement of any kind. After 30 minutes, the mice were returned to their original cages, and the number of marbles buried at a depth more than or equal to 75% of the bedding material was counted.

#### OF test.

Mice were transported to the testing room, or ideally an antechamber, and left undisturbed for 30 minutes before the test. The open field arena was divided into a peripheral zone measuring 8 cm from the edge of the arena walls and a central zone covering approximately 40% of the total surface of the arena. Each mouse was placed in the middle of a peripheral zone of the arena facing the wall and allowed to explore the apparatus freely for 20 minutes. The procedure room was maintained in silence without any kind of movement. At the end of the 20-minute run, the data were analyzed blindly. The time spent in the central zone (seconds) was recorded from the video.

### Gut microbiota analyses

Using our well-established 16S rRNA sequencing with the MiSeq Illumina platform and bioinformatics to analyze gut microbiota composition (55, 57–61), we determined the differences in old versus young feces. The genomic DNA was extracted using the QiaAmp PowerFecal Pro kit (QIAGEN). Primers 515 F (barcoded) and 806 R were used to amplify the V4 region of bacterial 16S rDNA (62), and we purified and quantified PCR products with AMPure magnetic purification beads (Agencourt) and Qubit-3 fluorimeter, respectively. The DNA libraries were prepared, and an equal amount (8 pM) of the amplicons was used for sequencing on an Illumina MiSeq sequencer (using MiSeq reagent kit v3). The sequences were de-multiplexed, quality filtered, clustered, and analyzed using the Quantitative Insights into Microbial Ecology and R-based analytical tools, as standard in our laboratory (55, 57–62).

### Metabolomics analysis

Fecal samples were extracted using a previously described method (63) with slight modifications (8, 64). The extracted water samples were mixed with phosphate buffer, so the final solutions contained 10% D_2_O with 0.1 M phosphate buffer (pH = 7.4) and 0.5 mM trimethylsilylpropanoic acid (TSP). NMR experiments were conducted on a Bruker Ascend 400 MHz, high-resolution NMR using a 1D first increment of a NOESY (noesygppr1d) with water suppression and a 4-second recycle delay. All NMR spectra were phased and referenced to TSP in TopSpin 4.06 (Bruker Biospin). The NMR peaks were extracted using Amix 4.0 (Bruker Biospin) with a previously reported automatic bucketing method to minimize peak splitting. Metabolite identification was carried out using Chenomx 8.6. Total intensity normalization was applied before further data analysis. Semiquantification of SCFAs was done by converting parts per million (ppm) values to μmol/g in feces, multiplying ppm values by 1,000 and dividing by molecular mass based on fixed amount (100 mg) of sample used for NMR detection.

### Gene expression using real-time PCR

Total RNA was extracted from frozen tissues, enteroids, and cells using a RNeasy Mini Kit following the manufacturer’s protocol (QIAGEN). cDNA was synthesized using a High-Capacity cDNA reverse transcription kit. Gene expression was quantified using an ABI 7500 real-time PCR, PowerUp SYBR Green Master Mix, and specific mouse and human primers (listed in Supplemental Tables 1 and 5). Relative gene expression was analyzed using the ^ΔΔ^CT method and normalized with 18S as an internal housekeeping control using standard protocols established in our lab (8, 47, 53, 65).

### Histochemical analyses for tissues and cell cultures

Intestinal tissues were collected, washed with PBS, fixed in 10% formalin, and sliced 5 μm thick, and the resulting sections were stained with H&E. Goblet cells were stained using an Alcian blue/PAS kit following the manufacturer’s instructions and counted as unstained vacuoles in the H&E immunohistochemistry slides. Mucus staining in the goblet cell line, CMT93 cells, was performed in a 12-well cell culture plate using the Alcian blue/PAS kit. All the CMT93 cells’ images were captured at original magnification, 4×, and mouse tissue histology was captured at original magnification, 20×, using an AmScope microscope. The goblet cell counts in ileum and colon tissue and PAS^+^ staining area percentage were quantified by FIJI software.

### Markers of systemic leaky gut and inflammation assays

LBP (Hycult Biotech) and sCD14 (R&D Systems) concentrations were measured in mouse serum to determine systemic leaky gut, while IL-6 and TNF-α concentrations were measured in serum to determine systemic inflammation using ELISA kits (R&D Systems).

### Fecal mucin content

Fecal mucin content for each mouse was measured in triplicate using an ELISA kit (Cosmo BioCo. Ltd) following the manufacturer’s protocol.

### Butyrate kinase and butyryl-CoA:acetate CoA transferase gene expression in feces

After total DNA was extracted from the mouse feces, its *buk* and *but* gene expression was measured by real-time quantitative PCR (see Supplemental Table 6) (47, 66).

### Preparation of FCM

FCM was prepared as described (8, 67). In brief, snap-frozen feces were ground in liquid nitrogen using a mortar and pestle. The fine powder was suspended in cold DMEM at a concentration of 100 mg fecal content in 100 mL of media. The suspension was shaken at 200 rpm for 1 hour at 4°C and filtered twice through a sterile 0.45 μm pore nylon membrane and twice through a sterile 0.22 μm nylon membrane syringe. The resulting FCM at 1:40 dilution was used to challenge the cells and enteroids at a concentration of 2.5 mg/mL.

### Enteroid development

The small intestinal organoids were prepared as described (56). In brief, the entire small intestine of 6- to 8-week-old B6 mice was washed with precooled Dulbecco’s PBS (DPBS), cut lengthwise, and fragmented into 2 mm pieces. These tissue fragments were washed, digested with trypsin, and filtered through a 70 μm cell strainer 2 times. The intestinal crypt-containing pellets were resuspended in cold IntestiCult Organoid Growth Medium containing 50 μg/mL gentamicin. Matrigel Matrix was added, and 50 μL of the mixture was pipetted slowly into the center of a prewarmed 24-well culture plate to form a dome. The plate was immediately incubated at 37°C and 5% CO_2_ for 30 minutes to allow the Matrigel to set, and 500 μL of IntestiCult Organoid Growth Medium was added to each well. To maintain the cultures, the IntestiCult Organoid Growth Medium was changed 3 times per week. The developed organoids were challenged with FFAR2 inhibitor (1 μM CATPB) and FFAR3 inhibitor (1 μM pertussis toxin), on day 10 for 2 hours, and then these enteroids were challenged with young and old FCM (2.5 mg/mL) and butyrate (6 μM). The organoids remained in these solutions for 48 hours and were harvested on the 12th day for gene expression analyses. Each experiment was conducted 3–4 times with 3 replicates each.

### Cell culture

Human HT29 cells and mouse CMT93 were purchased from American Type Culture Collection. The HT29 cells (passages 10–15) and CMT93 cells (passages 6–14) were maintained in 4.5 g/L d-glucose with l-glutamine DMEM supplemented with 10% FBS, 100 U/mL penicillin, and 100 U/mL streptomycin. The CMT93 cells were grown for 48 hours, then treated with young and old FCM (2.5 mg/mL), acetate (10 μM), propionate (6 μM), butyrate (6 μM), FFAR2 inhibitor (1 μM CATPB), and FFAR3 inhibitor (FFAR3 siRNA, Thermo Fisher Scientific) for 12 hours. The acetate, propionate, and butyrate concentrations used here are considered close to physiological concentration in the gut, as well as tolerable by cells and enteroids. Fully differentiated 21-day HT29 cells were used for young and old FCM treatment (2.5 mg/mL) for 8 hours to measure the TEER and FITC diffusion assay. The cells were washed with DPBS before harvesting.

### Measurements of TEER cell monolayers

HT29 cells were seeded on an apical chamber made of polyester membrane filters (0.4 μm pore size) in 12-well Transwell plates at a density of 3 × 10^5^/well. The culture media from both the apical and basolateral compartments were changed every 2 days. The cells were allowed to fully differentiate for the next 21 days. The fully differentiated cells were then challenged with FCM for 8 hours, continuously measuring TEER values using an EVOM^2^ Epithelial Voltohmmeter according to the manufacturer`s instruction. The blank inert resistance value (the insert with only culture media) was subtracted from the measured resistance value of each sample, and the final resistance (reported in ohm × cm^2^) was calculated by multiplying the sample resistance by the area of the membrane (8).

### FITC-dextran permeability assay in cell monolayers

The HT29 cell Transwell plates were prepared using the same method as in the TEER experiments. On the 21st day, the HT29 cell monolayer was treated with FCM, and 4 kDa FITC-dextran solution (1 mg/mL) was added on the apical (upper) side of the monolayers. The 4 kDa FITC level of the basolateral side was determined using a fluorescent 96-well plate at a 485 nm excitation and a 525 nm emission wavelength using a fluorescence 96-well plate reader. Values were normalized using a standard FITC curve as described in our paper (52). The assay was conducted 3 times in triplicate.

### Statistics

Different data sets were analyzed using 2-tailed *t* test and 1- or 2-way ANOVA, as appropriate. All figures’ data are presented as mean ± SEM. The bacterial abundance of old and young microbiome was compared using the unpaired 2-tailed Student’s *t* test. Differences in β-diversity were tested by permutational multivariate analysis of variance, a permutation-based multivariate analysis of variance to a matrix of pairwise distance to partition the inter- and intragroup distances. Random forest analysis and PCA were analyzed in R (version 3.6.0, https://www.r-project.org/) using the packages randomForest, ggplot2, caret, psych, ggbiplots, nnet, and devtools. Hierarchical clustering and heatmaps of the top 50 genera based on abundance were constructed using the pheatmap and ggplots packages of R v6.0. LEfSe was used to identify unique bacterial taxa that drive differences in old versus young fecal samples (Galaxy server; https://huttenhower.sph.harvard.edu/galaxy/) (68). The alpha parameter significance threshold for the Kruskal-Wallis as well as the Wilcoxon signed-rank test, which were implemented among classes, was set to 0.01. The logarithmic LDA score cutoff was set to 3, and the strategy for multiclass analysis was set to “all-against-all.” Welch’s *t* test was applied to analyze the statistical significance of metabolites using Amix 3.9 (Bruker Biospin), and an FDR was applied to control the family-wise error. Hierarchical clustering was done and heatmaps were constructed based on the average linkage on Euclidean distance and depict the patterns of abundance and log values of metabolites and their correlation with gut permeability, inflammation, and tight junction markers. Graphs were constructed in R v6.0 using the pheatmap and ggplots packages, as well as GraphPad v9. The Pearson’s correlation was calculated between respective groups in IBM SPSS v27, and clustering correlation heatmaps were constructed using the pheatmap package, R v6.0. *P* < 0.05 was considered statistically significant for all analyses unless otherwise specified.

### Study approval

All animal experiments and procedures were approved by the IACUC of the USF.

### Data availability

Supporting Data Values are available in the metadata XLS file.

## Author contributions

SPM, SJ, BW, and SW conducted overall experiments and data analysis and wrote the first draft; BCM, JYL, CVB, LJ, and JP helped in microscopy and metabolomics analyses; and ST, BTL, and SGR edited the manuscript and participated in intellectual discussions. HY conceived the idea, arranged the resources and funding, and wrote, edited, and finalized the manuscript.

## Supplementary Material

Supplemental data

Supporting data values

## Version 1 02/08/2024

Electronic publication
