# Supplementary material for: Abnormalities in microbiota/butyrate/FFAR3 signaling in aging gut impair brain function
Source: JCI Insight. 2024 Feb 8;9(3):e168443. doi: 10.1172/jci.insight.168443 (PMC10967378; doi:10.1172/jci.insight.168443)
Supplement: Supplemental data [file jciinsight-9-168443-s008.pdf]

# Supplementary Figures

**Mishra et al**

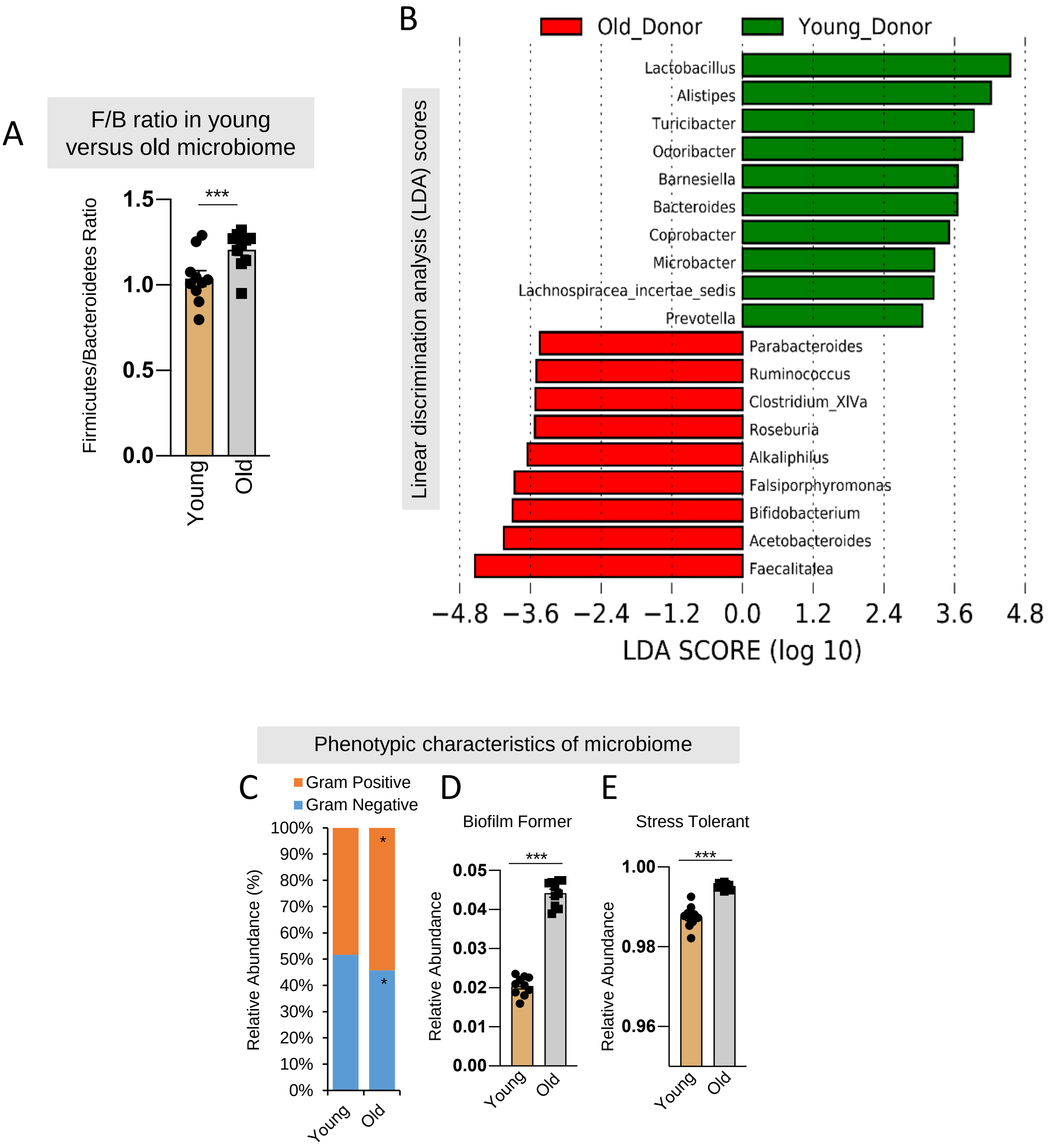


**Supplementary Figure S1.** (**A**) The Firmicutes versus Bacteroidetes (F/B) ratio was significantly increased in old mice compared to young mice. (**B**) The LDA score of microbes that abundant in young (green) compared to old (red) and represent a biomarker ability. (**C-E**) The relative abundance of Gram positive and Gram negative (C) as well as biofilm former (D) and stress tolerant (E) microbes in young and old gut. All values represent the mean of 5-10 animals in each group, and error bars represent the standard error of means. Statistical significance was determined using a t-test, and the p-value ***p<0.001 is statistically significant.

## Mishra et al Supplementary Figure S2

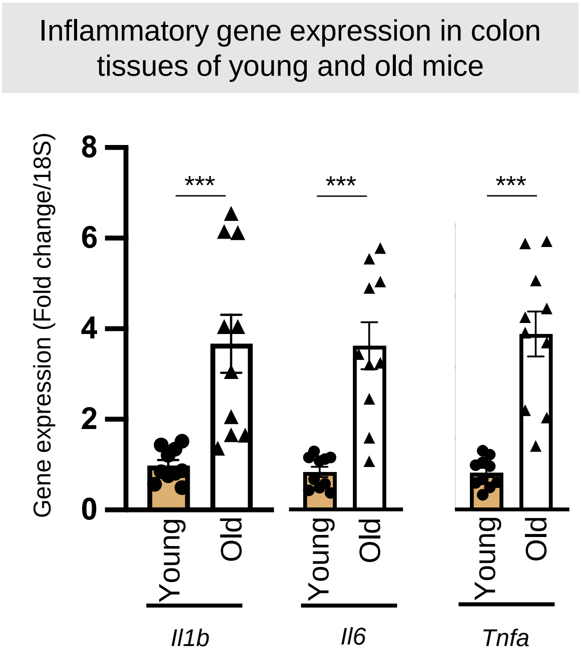


**Supplementary Figure S2.** The expression of inflammatory genes (*Il1b, Il6*, and *Tnfa*) significantly increased in the colon of old mice compared to young mice. All values represent the mean of 5-10 animals in each group, and error bars represent the standard error of means. Statistical significance was determined using a t-test, and the p-value ***p<0.001 is statistically significant.

## Mishra et al Supplementary Figure S3

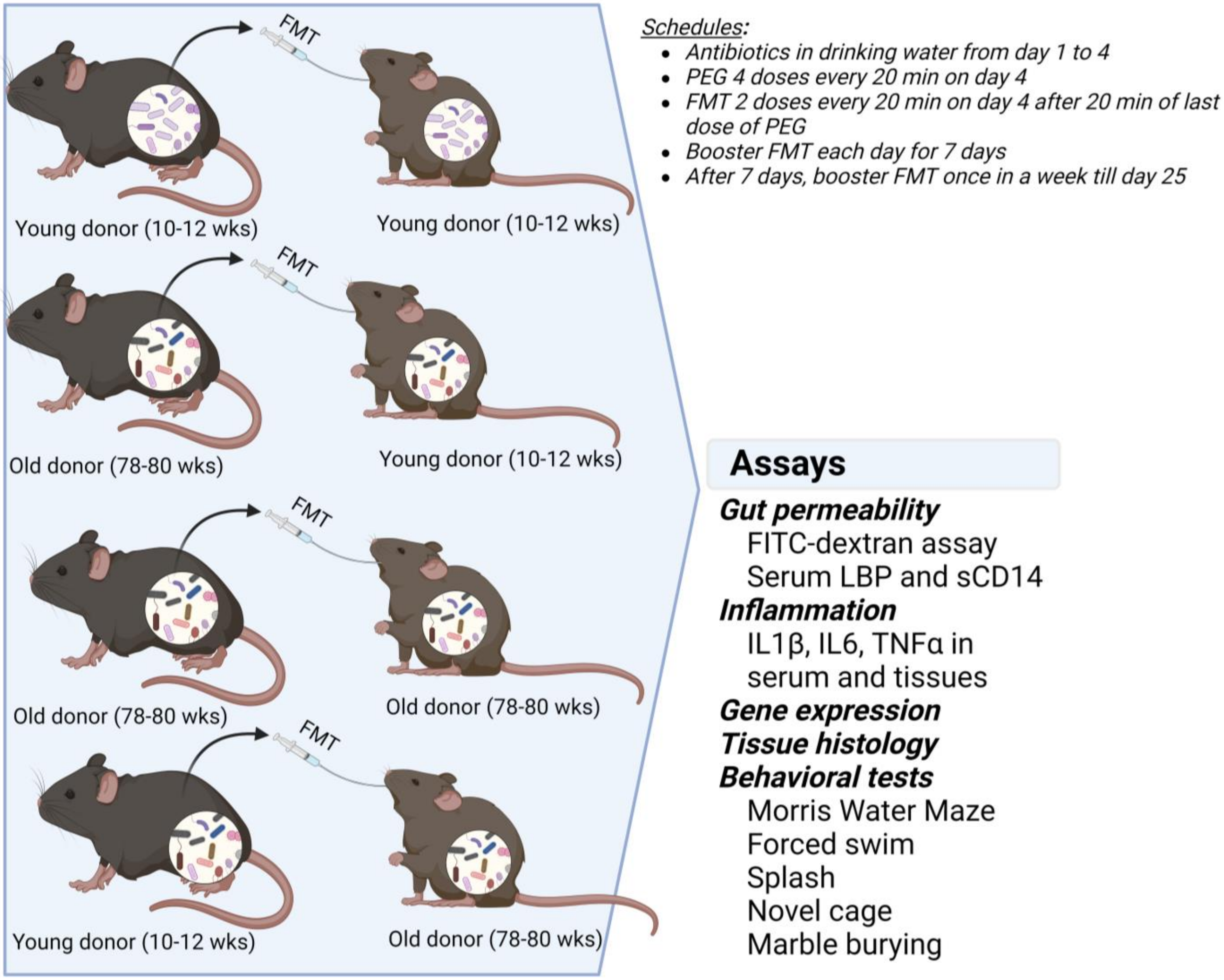


**Supplementary Figure S3.** Schematic presentation of the fecal microbiome transplantation (FMT) experiment and the schedules for young to young, old to young, old to old, and young to old, along with the assays performed.

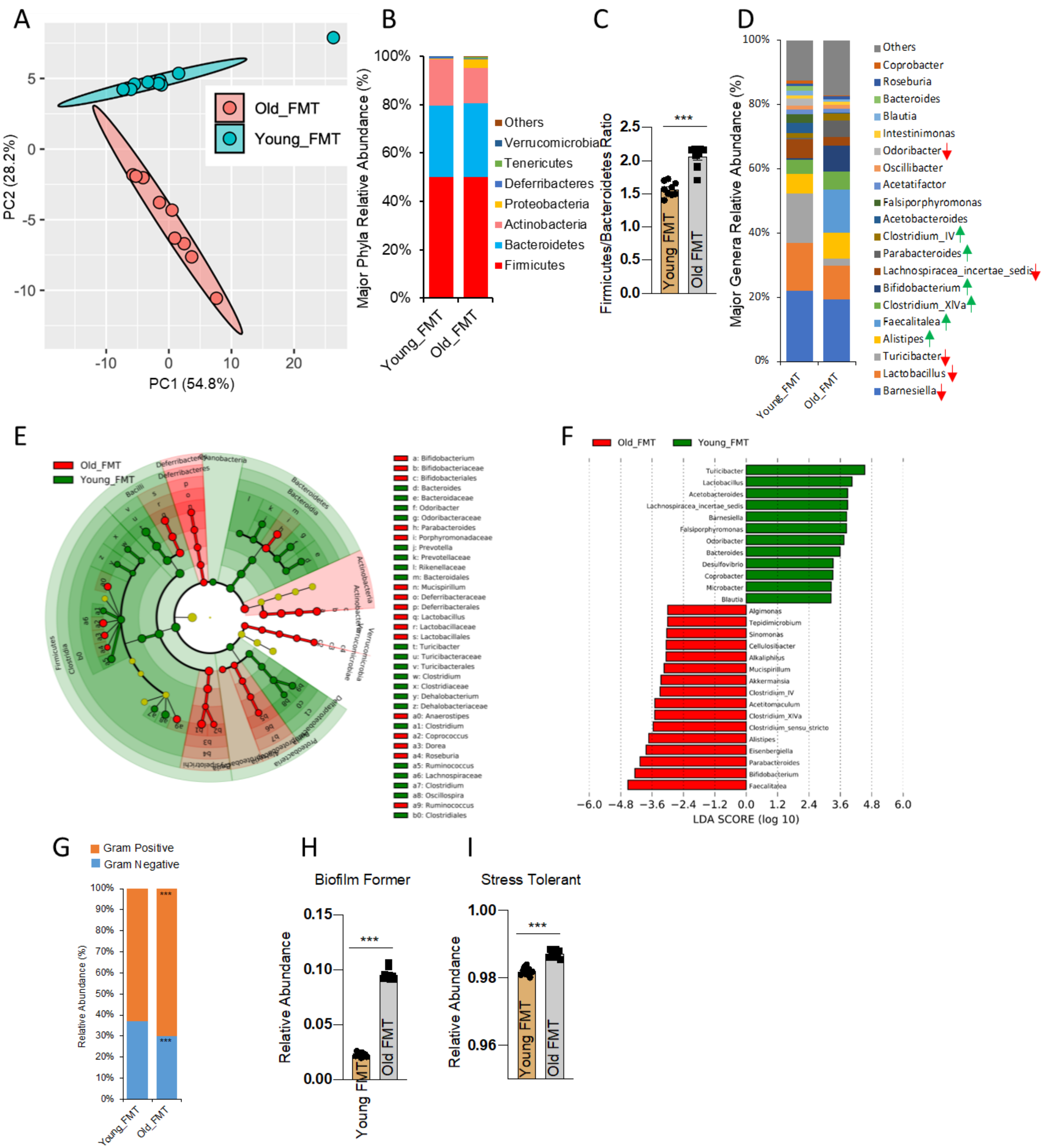


**Supplementary Figure S4**. (**A**) The microbiome's β-diversity was significantly different between old and young FMT recipient mice and was similar to that of their respective donors. (**B,C**) The abundance of major phyla (B), specifically the Firmicutes/Bacteroidetes ratio (C), was significantly distinct in older FMT recipients compared to younger recipients. D) The abundance of major genera differed significantly between old and young FMT recipient feces. (**E,F**) Similarly, the cladogram (E) and LDA scores (F). (**G-I**) Abundance of higher gram-negative bacteria (G), biofilm former (H) and stress tolerant (I) in old FMT mice as compared to Young FMT mice. All values represent the mean of 5-10 animals in each group, and error bars represent the standard error of the means. Statistical significance was determined using a t-test and/or ANOVA, with a p-value of ***p<0.001, indicating statistical significance.

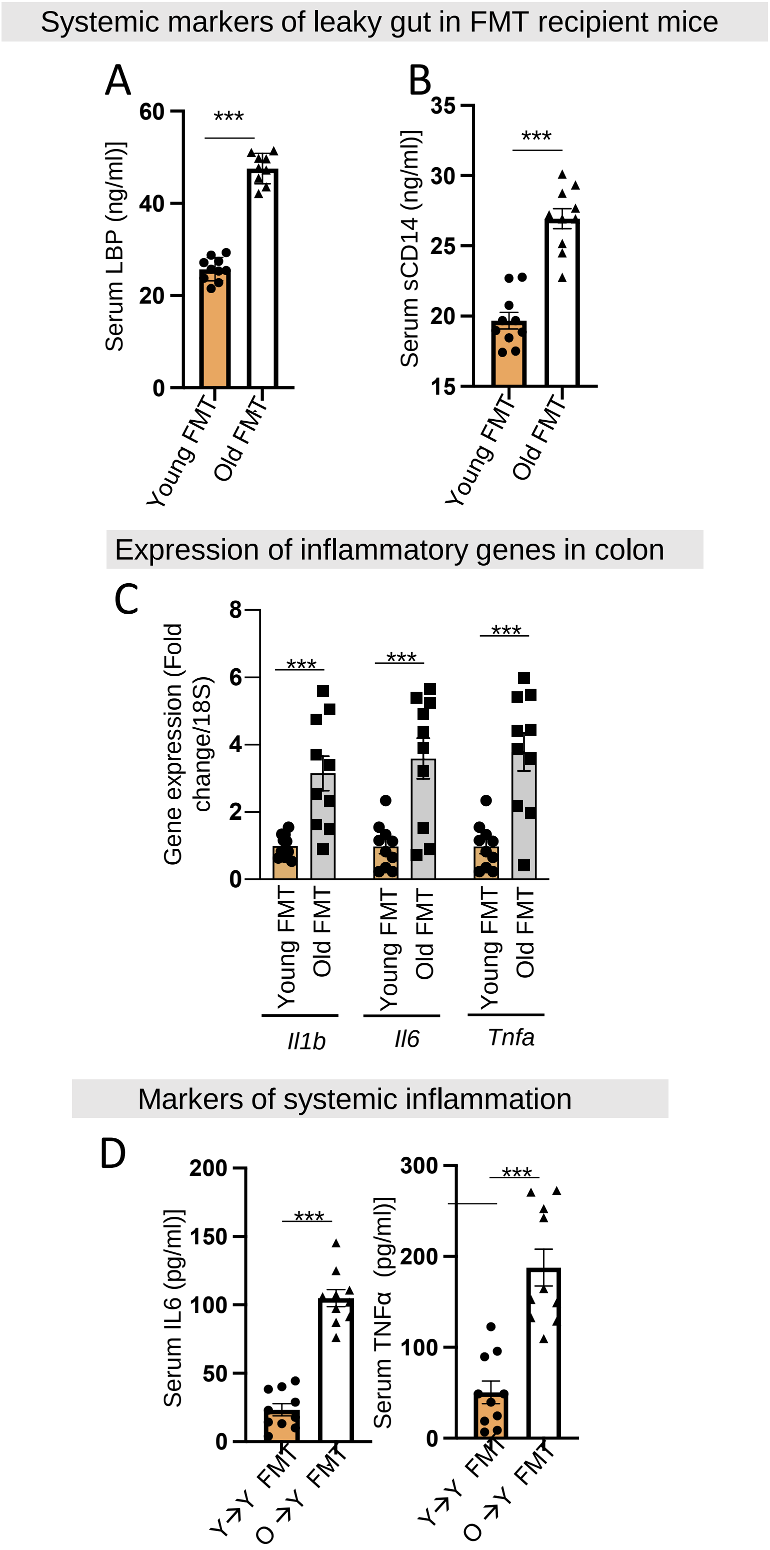


**Supplementary Figure S5**. (**A,B**) The levels of systemic markers of leaky gut (LBP and sCD14) significantly increased in 10-12 weeks old mice who received old FMT compared to those who received young FMT. (**C**) The expression of inflammatory genes (*Il1b*, *Il6*, and *Tnfa*) significantly increased in old FMT recipients compared to young FMT recipients. (**D**) Similarly, the old FMT recipient mice showed higher systemic inflammatory markers like IL6 and TNFα compared to the levels in young FMT recipients. All values represent the mean of 5-10 animals in each group, and error bars represent the standard error of the means. Statistical significance was determined using a t-test and/or ANOVA, with a p-value of ***p<0.001, indicating statistical significance.

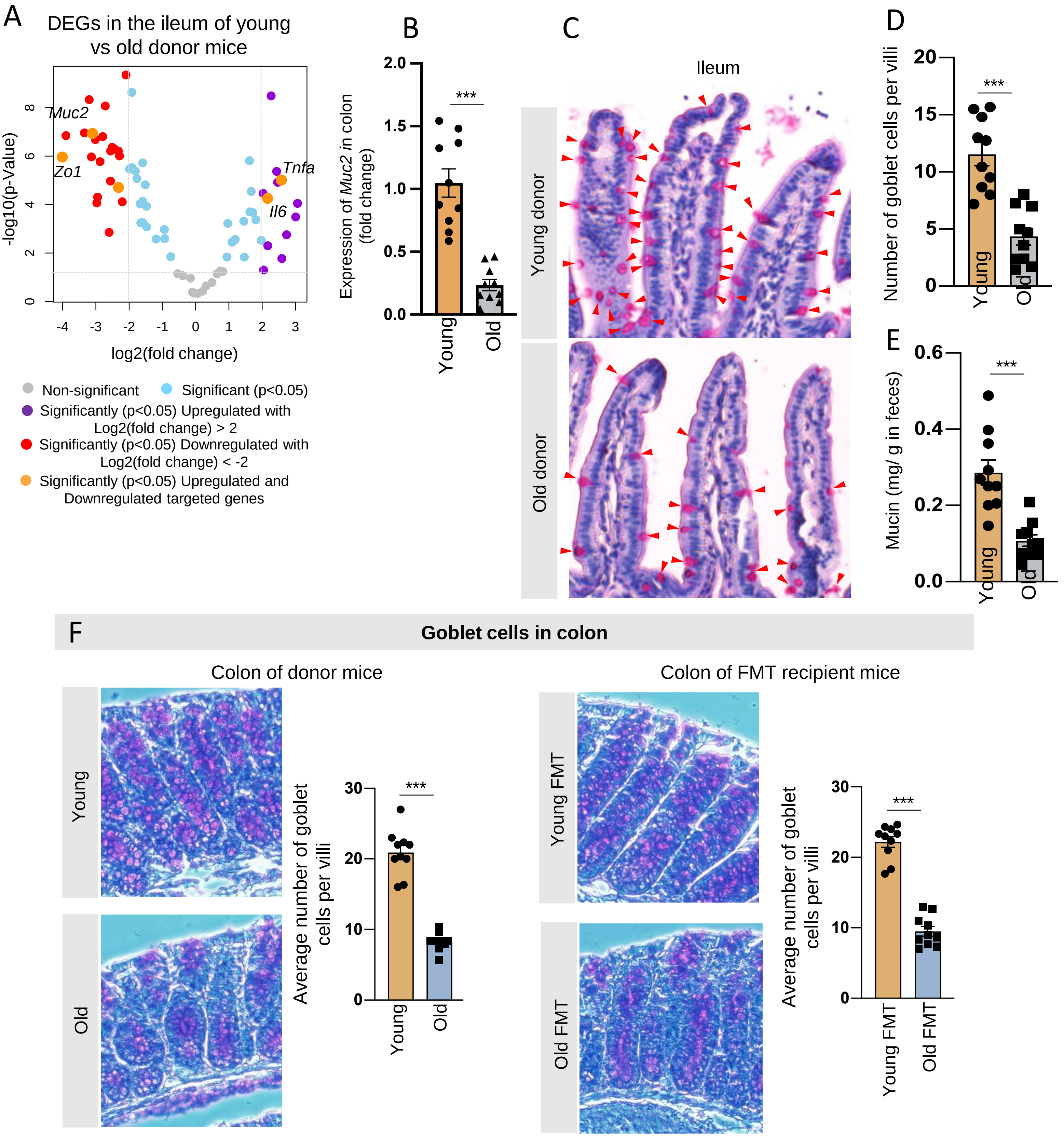


**Supplementary Figure S6**. (**A**) Volcano graph of differentially expressed genes in the intestines of old and young mice. (**B**) Muc2 expression in the ileum of old and young donor mice. (**C,D**) Small intestine histology (H&E staining) shows fewer goblet cells (white bubble-like structures indicated by red arrows) in old mice than in young mice. (**E**) Fecal mucin content in older mice decreased compared to young mice. (**F**) The goblet cell population in the colon was found to be lower in older mice than in younger mice, and old FMT recipients also showed decreased goblet cells compared to young FMT recipients. Number of goblet cells per villi was measured by FIJI. All values represent the mean of 5-10 animals in each group, and the pictures are representative of 5-10 animals. Five pictures were taken of each animal's tissue sections. Statistical significance was determined using a t-test, and the p-value ***p<0.001 is statistically significant.

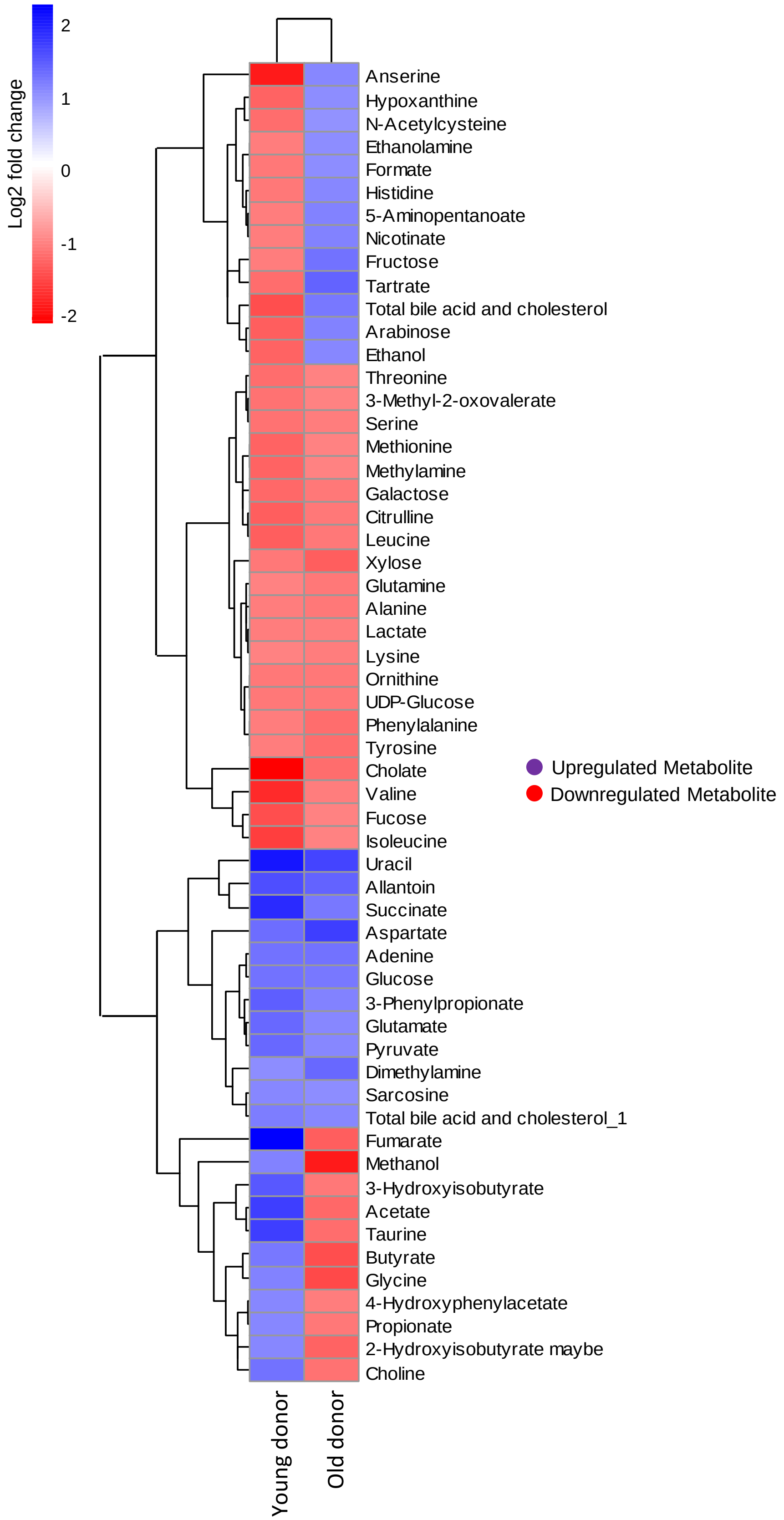


**Supplementary Figure S7**. Hierarchical differential clustering analyses reveal significant differences in metabolites between old and young gut (ileum). All the values represent the mean of 5-10 animals in each group, and error bars represent the standard error of means. Statistical significance was determined using t-test.

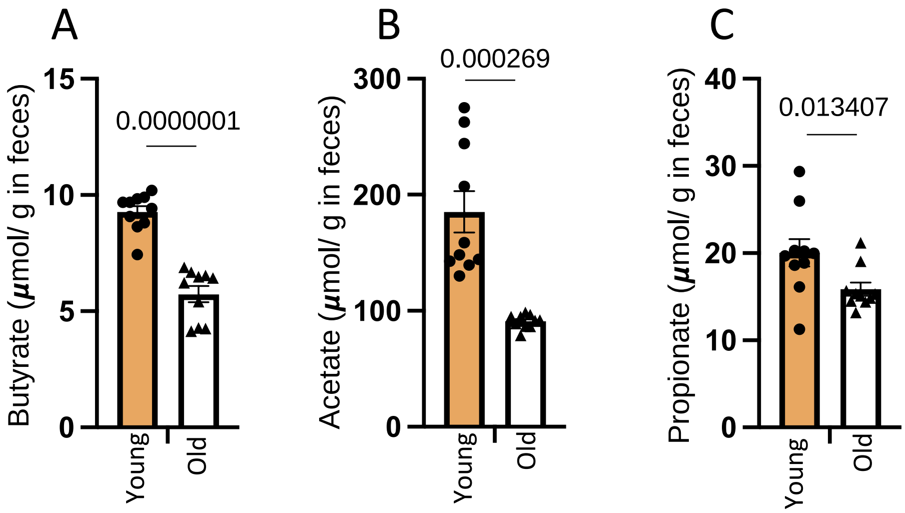


**Supplementary Figure S8.** Comparing the abundance of three SCFAs—(**A**) Butyrate, (**B**) Acetate, and (**C**) Propionate —in old to young feces shows the greatest decrease (based on p-values) in butyrate. Values represent the mean of 5-10 animals in each group, and error bars represent the standard error of means. Statistical significance was determined using t-test, and the actual p-values are presented in the graphs.

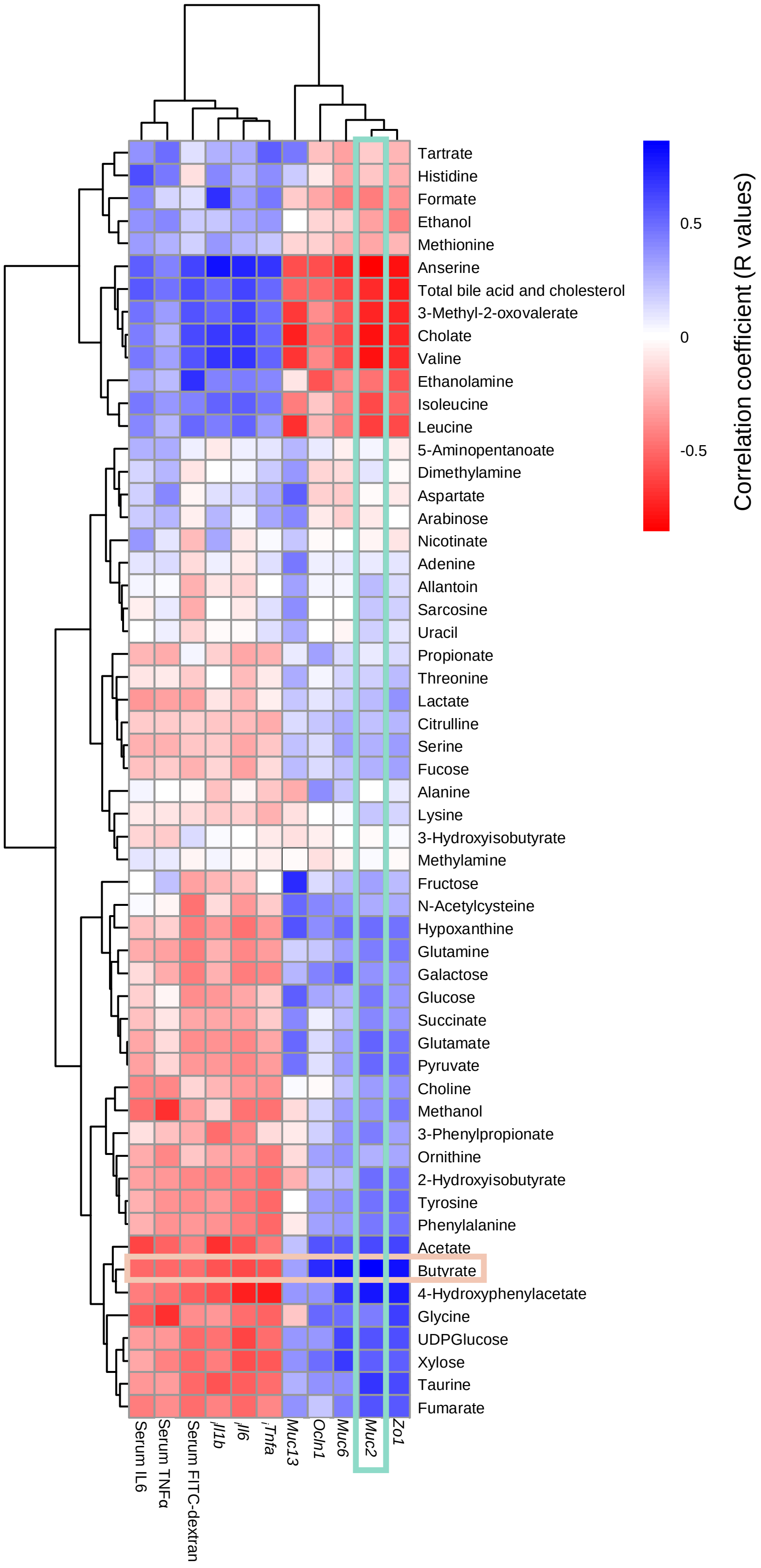


**Supplementary Figure S9.** Heatmap of Pearson correlation values among fecal metabolites with mucin genes (*Muc2, Muc6, Muc13*), tight junction protein genes (*Zo1, Ocln1*), leaky-gut markers (FITC-dextran permeability [indicated as "FITC"], and inflammation (both intestinal [indicated by "i"] and systemic [serum IL6 and TNFα]) revealed unique clustering. The highest correlation was between butyrate and *Muc2* gene expression.

**Mishra et al Supplementary Figure S10**

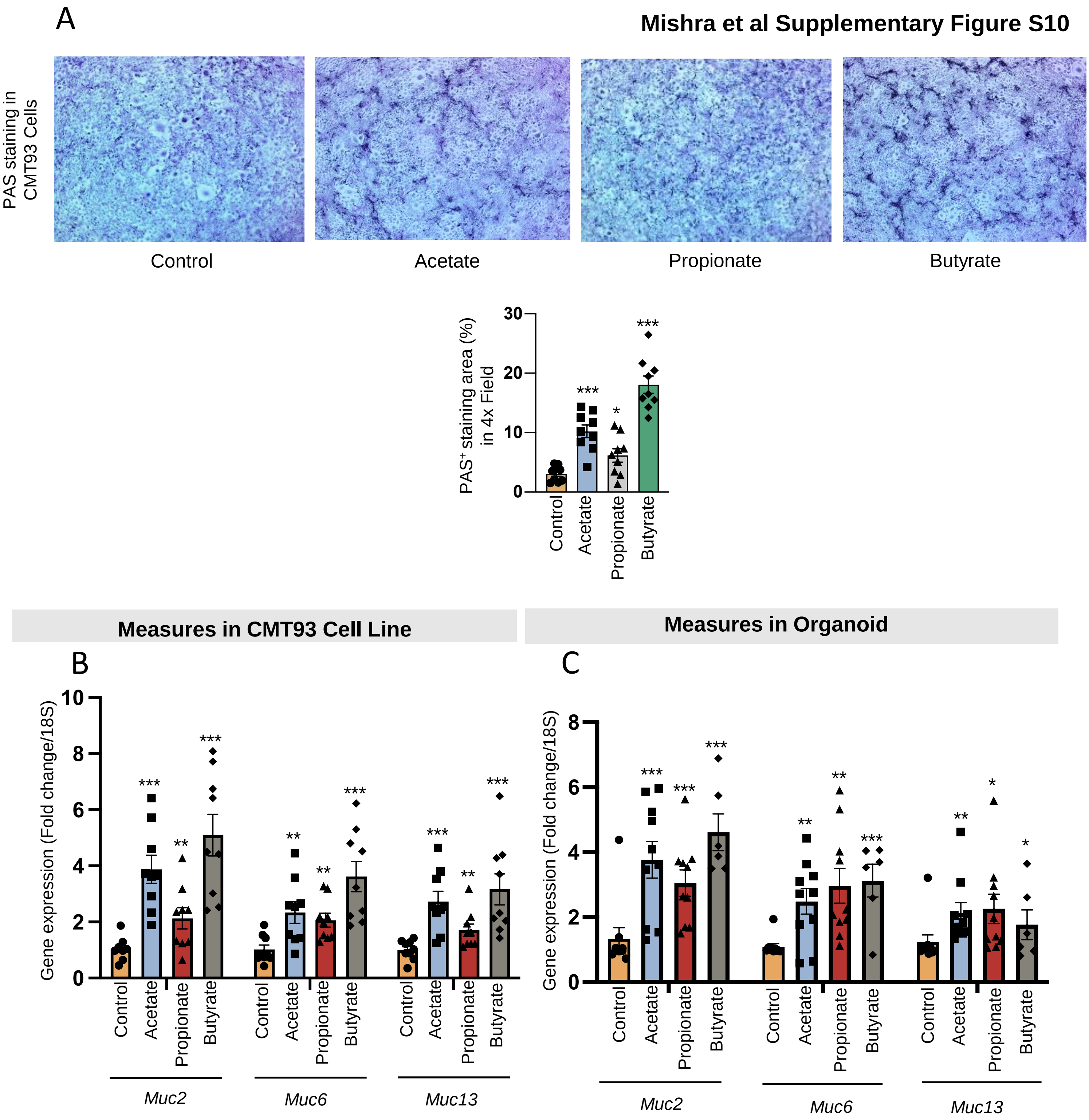


**Supplementary Figure S10.** Acetate (10μM), propionate (6μM), and butyrate (6μM) treatment enhances mucin production (indicated by increased blue of Periodic Acid–Schiff [PAS] staining) and increased PAS positive ($PAS^+$) area percentage (%) quantification measured by FIJI (A) and *Muc2*, *Muc6,* and *Muc13* gene expression in both CMT93 cells (B) and enteroids (C). All the values represent the mean of 3-4 independent replicates in cells and organoid cultures for each group, and error bars represent the standard error of means. Statistical analyses were performed using one- and/or two-way ANOVAs, and p-values *p<0.05, **p<0.01, and ***p<0.001 are statistically significant.

**Mishra et al Supplementary Figure S11**

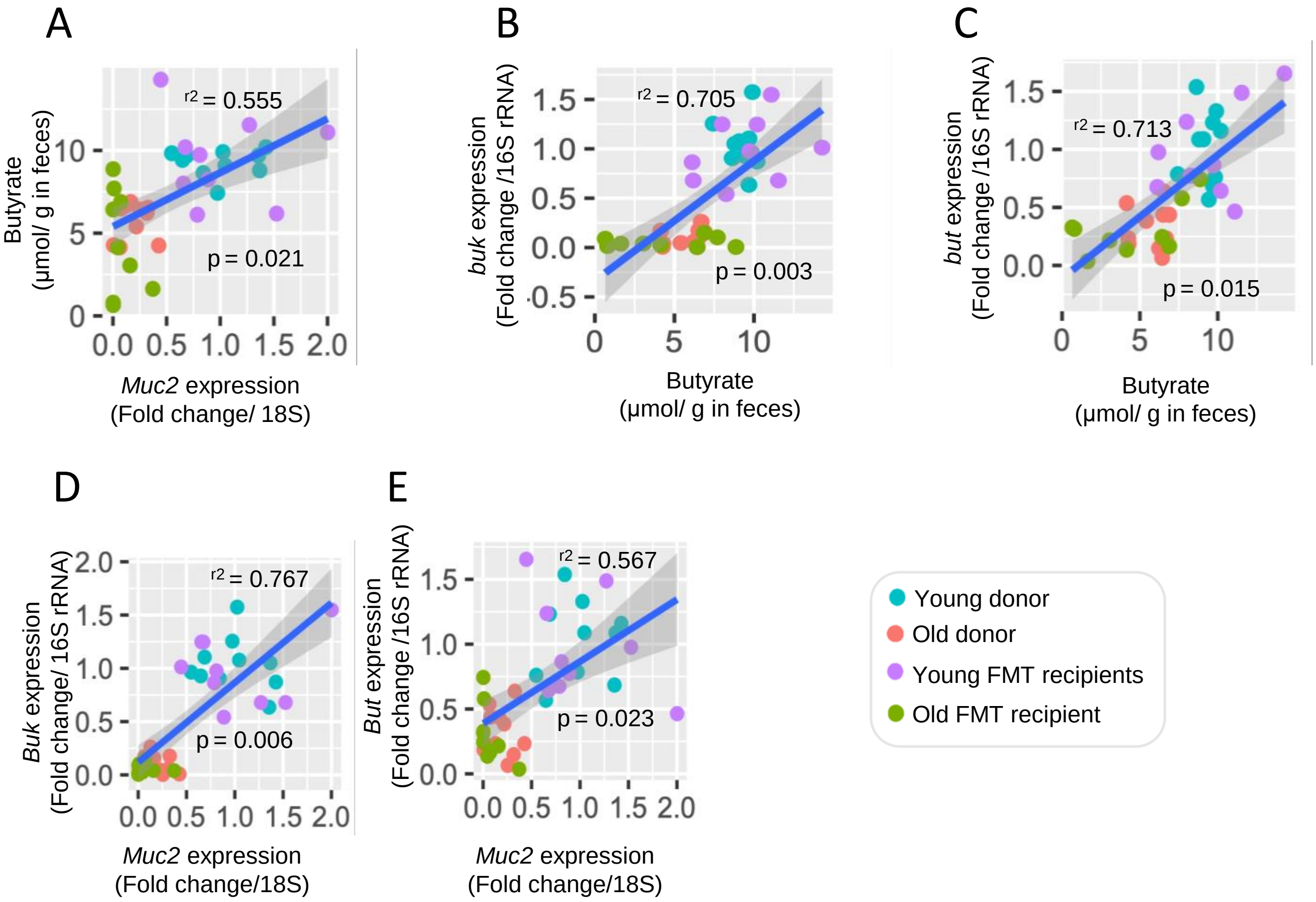


**Supplementary Figure S11.** (**A-E**) Multivariate analyses correlating butyrate with *Muc2* (A), *buk* (B), and *but (C)* genes as well as *Muc2* expression with *buk* and *but* genes (D,E) in young donors, old donors, young FMT recipients, and old FMT recipients. Analyses are done using Pearson correlation analysis and $r^2$ values plotted using r-scripts. Values represent the mean of 3-4 independent replicates in cells cultures for each group, and error bars represent the standard error of means. Statistical analyses were performed using t-test and/or ANOVA, as applicable, and the actual p-values are presented in the graphs.

**Mishra et al Supplementary Figure S12**

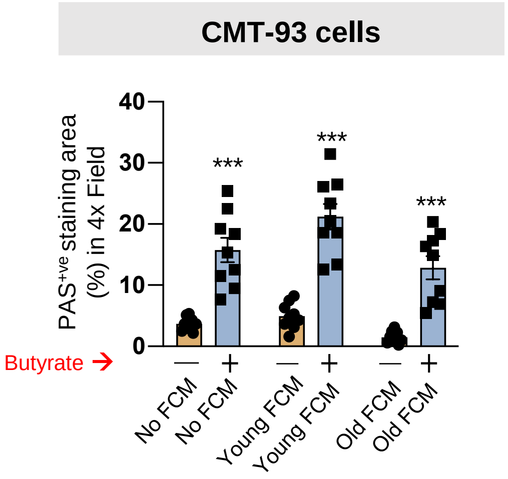


**Supplementary Figure S12**. Butyrate treatment increased PAS positive (PAS+) area percentage (%) quantification in CMT93 cells dampened by old FCM treatment measured by FIJI. Values represent the mean of 3-4 independent replicates in cells cultures for each group, and error bars represent the standard error of means. Statistical analyses were performed using t-test and p-values ***p<0.001 are statistically significant.

**Mishra et al Supplementary Figure S13**

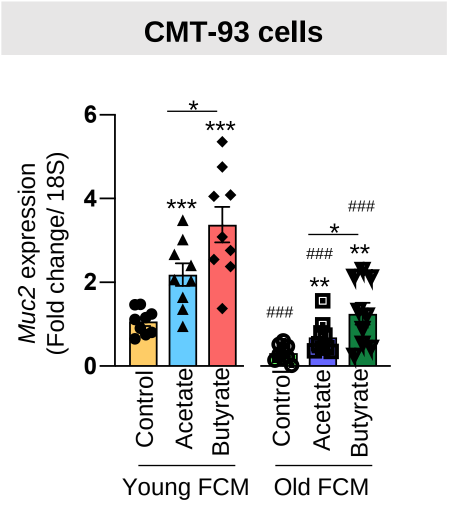


### t-Test between Young FCM vs Old FCM

**Supplementary Figure S13**. Both acetate and butyrate significantly increased the expression of *Muc2* expression in CMT93 cells treated with young and old FCMs, however, butyrate effects were significantly higher than acetate in both young and old FCM treated cells. Values represent the mean of 3-4 independent replicates in cells cultures for each group, and error bars represent the standard error of means. Statistical analyses were performed using t-test and p-values $^*p<0.05$, $^{**}p<0.01$, and $^{***}p<0.001$ are statistically significant.

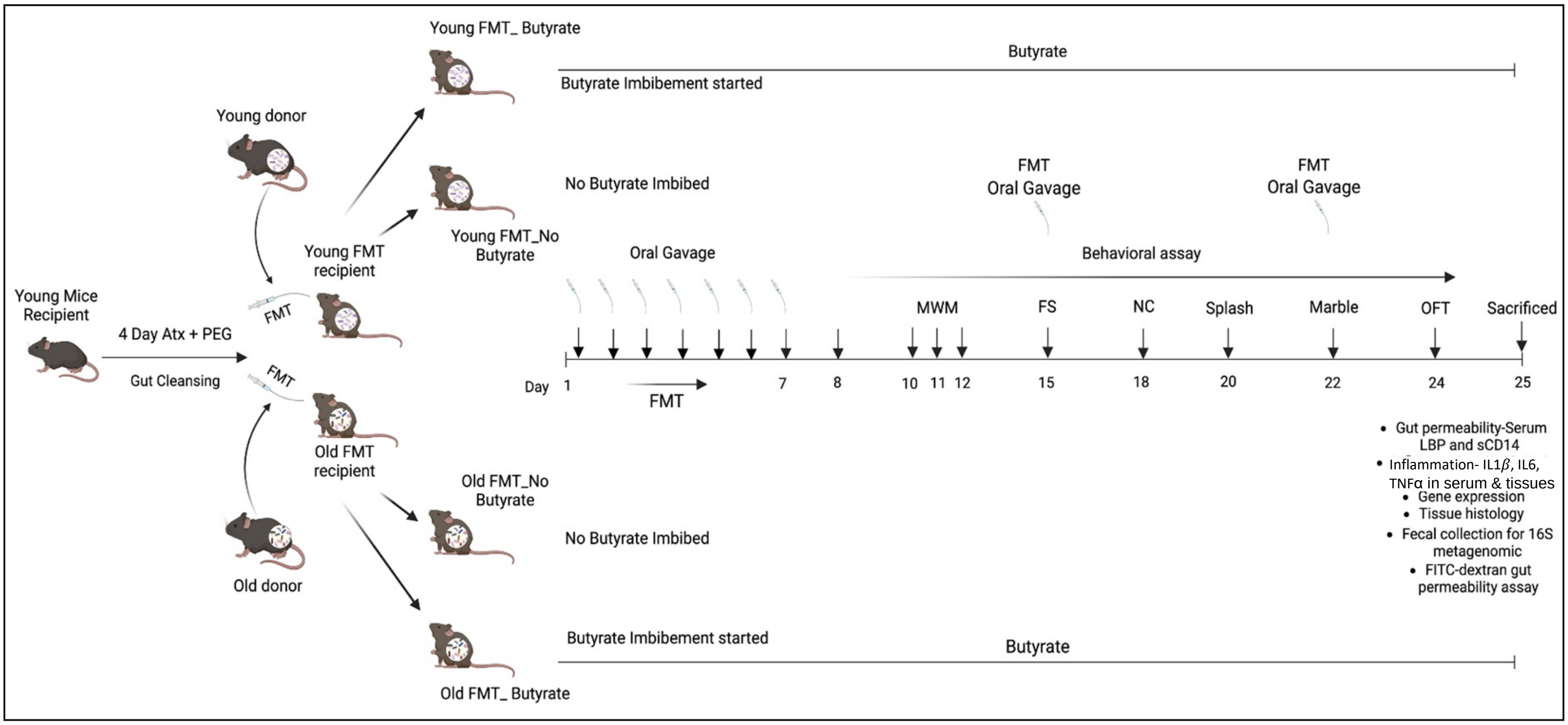


**Supplementary Figure S14.** Schematic of group randomization, gut cleansing, FMTs, treatments, and assays conducted to test the effects of butyrate in old FMT recipient mice compared to young FMT recipients, as data presented in main Figure 5D-M.

## Mishra et al Supplementary Figure S15

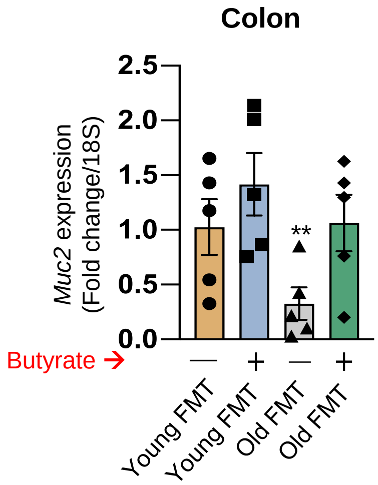


**Supplementary Figure S15.** Expression of *Muc2* in the colon of mice received old and young FMTs and treated with and without butyrate. All the values represent the mean of 5-8 animals for each group, and error bars represent the standard error of means. Statistical significance was determined using t-Test, and p-values **p<0.01 is statistically significant.

## Mishra et al Supplementary Figure S16

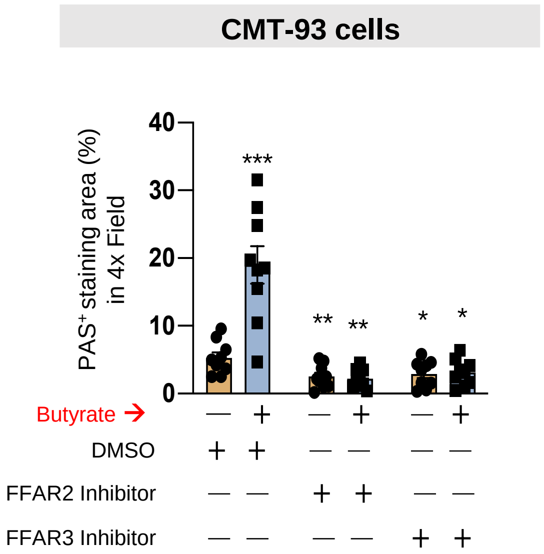


**Supplementary Figure S16**. Positive effect of butyrate increased in PAS positive ($PAS^+$) area percentage (%) quantification in CMT93 cells reduced by challenging with FFAR2 inhibitor and FFAR3 inhibitor measured by FIJI. Values represent the mean of 3-4 independent replicates in cells cultures for each group, and error bars represent the standard error of means. Statistical analyses were performed using t-test and/or ANOVA, as applicable, and p-values *p<0.05, **p<0.01, and ***p<0.001 are statistically significant.

*Ffar2/3* Mice study Design

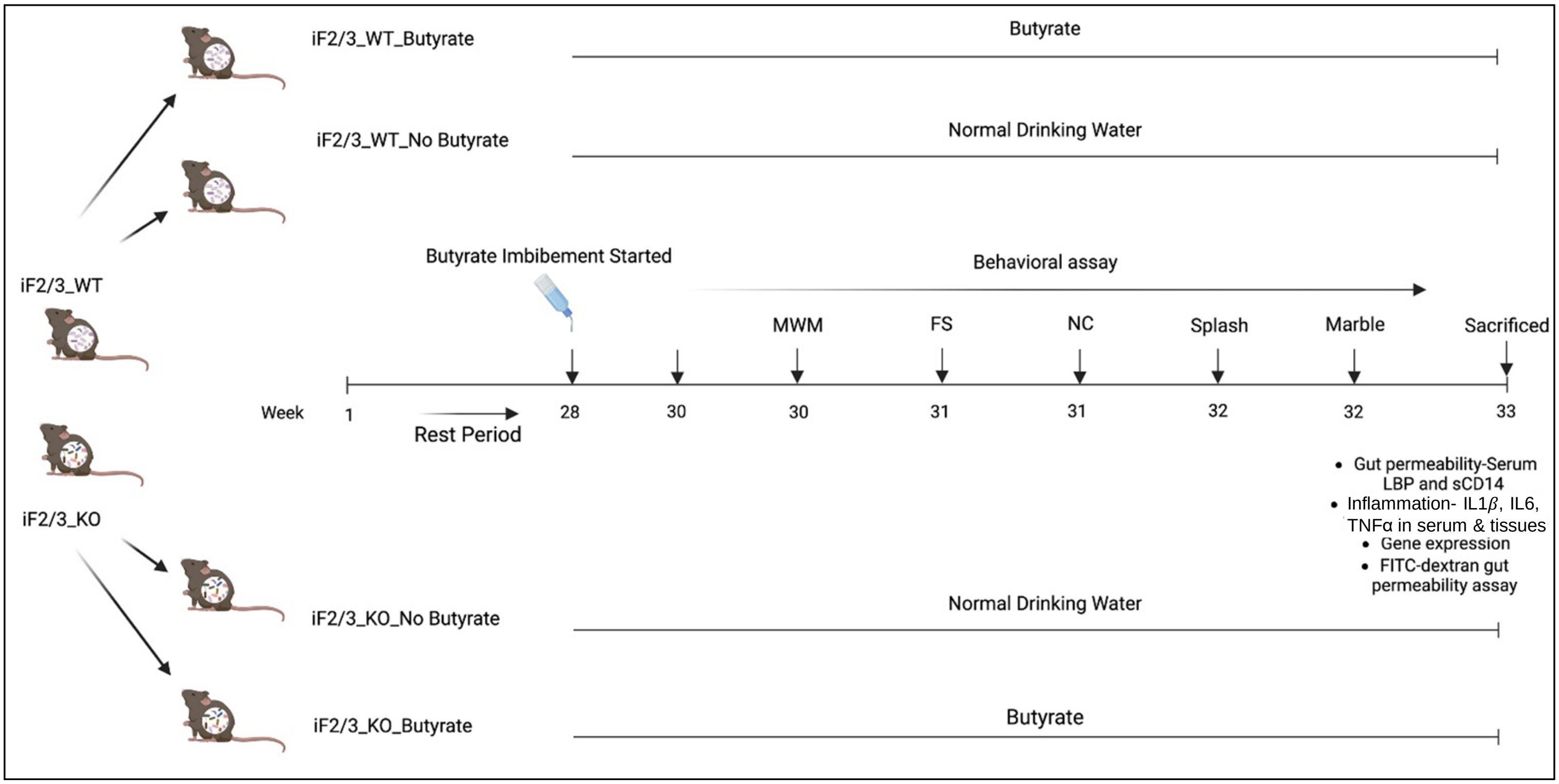


**Supplementary Figure S17**. Schematic of group randomization, gut cleansing, FMTs, treatments, and assays conducted to test the effects of butyrate in Villin-Cre driven intestine specific *Ffar2* (iF2) and *Ffar3* (iF3) knockout (KO) mice compared to their wildtypes (WT), as data presented in main Figure 7A-M.

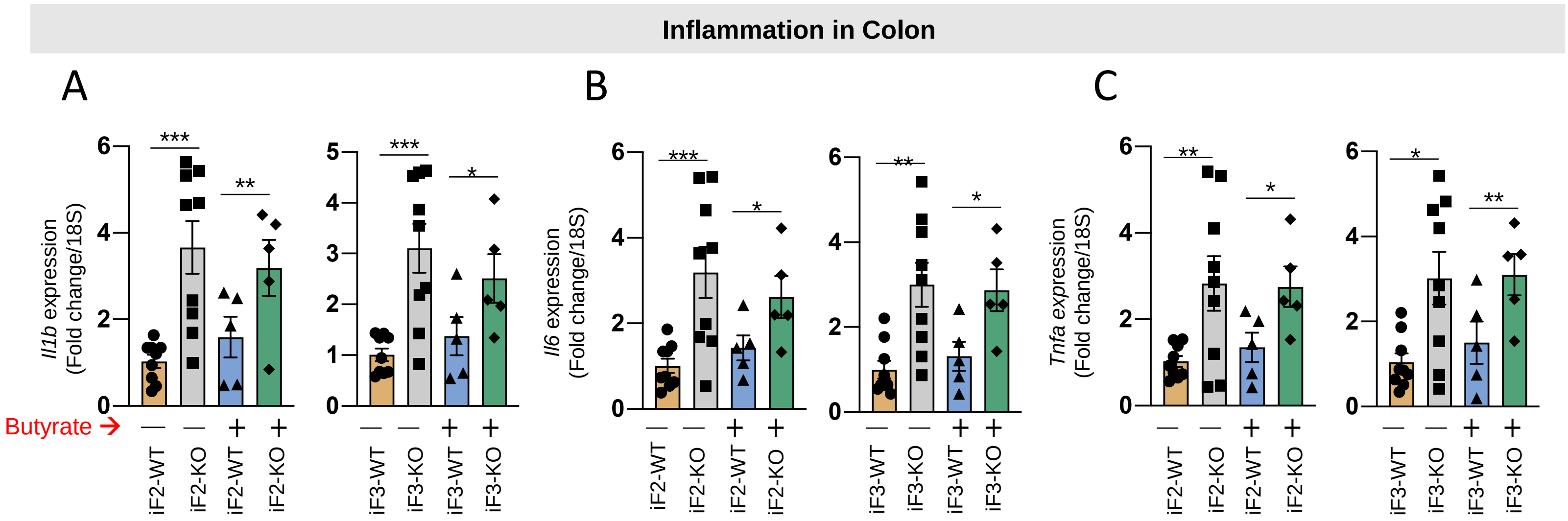


**Supplementary Figure S18**. The expression of inflammatory markers (*Il1b*, *Il6*, *Tnfa*) (A-C) significantly increased in the colon of 7-months old intestine-specific *Ffar2* (iF2) and *Ffar3* (iF3) knockout (KO) mice compared to their age- and sex-matched wildtype (WT) controls. All values represent the mean of 5-10 animals in each group, and error bars represent the standard error of the means. Statistical significance was determined using a t-test, as applicable, and p-values of *p<0.05, **p<0.01, and ***p<0.001 are statistically significant.

**Supplementary Table S1. Mouse key intestinal epithelial, neuronal, tight junction protein and inflammatory markers primer sequence**

| Sl. No. | Gene | Gene sequence (5`-3`) | GenBank Accession number/Ref. |
|---|---|---|---|
| 1 | ***Notch1*** | F: GATGGCCTCAATGGGTACAAG<br>R: TCGTTGTTGTTGATGTCACAGT | NM_008714* |
| 2 | ***Wnt3*** | F: CTCGCTGGCTACCCAATTTG<br>R: CTTCACACCTTCTGCTACGCT | NM_009521* |
| 3 | ***Ocln*** | F: TTGAAAGTCCACCTCCTTACAGA<br>R: CCGGATAAAAAGAGTACGCTGG | NM_008756* |
| 4 | ***Zo1*** | F: GCCGCTAAGAGCACAGCAA<br>R: GCCCTCCTTTTAACACATCAGA | NM_001163574* |
| 5 | ***Pcna*** | F: TTTGAGGCACGCCTGATCC<br>R: GGAGACGTGAGACGAGTCCAT | NM_011045* |
| 6 | ***Ccnd1*** | F: GCGTACCCTGACACCAATCTC<br>R: CTCCTCTTCGCACTTCTGCTC | NM_007631* |
| 7 | ***Kir67*** | F: ATCATTGACCGCTCCTTTAGGT<br>R: GCTCGCCTTGATGGTTCCT | NM_001081117* |
| 8 | ***Lyz1*** | F: GAGACCGAAGCACCGACTATG<br>R: CGGTTTTGACATTGTGTTCGC | PMID: 26159695# |
| 9 | ***Gp2*** | F: ACAGTGTCAACCATCTTGCTC<br>R: CCCGATTATAGTCAATGGCTGG | NM_025989* |
| 10 | ***Tff3*** | F: TTGCTGGGTCCTCTGGGATAG<br>R: TACACTGCTCCGATGTGACAG | PMID: 19185845# |
| 11 | ***Elf3*** | F: GCTGCCACCTGTGAGATCAG<br>R: GTGCCAAAGGTAGTCGGAGG | NM_001163131* |
| 12 | ***Spdef*** | F: AAGGCAGCATCAGGAGCAATG<br>R: CTGTCAATGACGGGACACTG | NM_013891* |
| 13 | ***Gfi*** | F: AGAAGGCGCACAGCTATCAC<br>R: GGCTCCATTTTCGACTCGC | NM_010278* |
| 14 | ***Math1*** | F: GAGTGGGCTGAGGTAAAAGAGT<br>R: GGTCGGTGCTATCCAGGAG | NM_007500* |
| 15 | ***Muc2*** | F: TTCGGCACGAGCAACTTTG<br>R: GGCAGGACACCTTGTCATTG | PMID: 32916121# |
| 16 | ***Muc6*** | F: CGGCTGCGTCTGTCCTAAG<br>R: GCATAGTCACATGGGCATTCCT | NM_181729* |
| 17 | ***Muc13*** | F: GATCTCTGCAACCCTAACCCC<br>R: TCCTTTCACACATGACGACAG | NM_010739* |
| 18 | ***Il10*** | F: GCTCTTACTGACTGGCATGAG<br>R: CGCAGCTCTAGGAGCATGTG | PMID: 32302292# |
| 19 | ***Tgfβ1*** | F: CTCCCGTGGCTTCTAGTGC<br>R: GCCTTAGTTTGGACAGGATCTG | NM_011577* |
| 20 | ***Il1β*** | F: GCAACTGTTCCTGAACTCAACT<br>R: ATCTTTTGGGGTCCGTCAACT | NM_008361* |

| Sl. No. | Gene | Gene sequence (5`-3`) | GenBank Accession number/Ref. |
|---|---|---|---|
| 21 | ***Il6*** | F: CCAAGAGGTGAGTGCTTCCC | PMID: 32302292# |
| | | R: CTGTTGTTCAGACTCTCTCCCT | |
| 22 | ***Tnfα*** | F: CCCTCACACTCAGATCATCTTCT | NM_013693* |
| | | R: GCTACGACGTGGGCTACAG | |
| 23 | ***Sis*** | F: GCTATCGCTCTTGTTGTGGTT | NM_001081137* |
| | | R: TTCCAGGACTAGGGGTTGAAG | |
| 24 | ***Call*** | F: TCCCACCACTGGGGATACAG | NM_009801* |
| | | R: CTCTTGGACGCAGCTTTATCATA | |
| 25 | ***Hes1*** | F: CCAGCCAGTGTCAACACGA | NM_008235* |
| | | R: AATGCCGGGAGCTATCTTTCT | |
| 26 | ***Casp3*** | F: ATGGAGAACAACAAAACCTCAGT | NM_009810* |
| | | R: TTGCTCCCATGTATGGTCTTTAC | |
| 27 | ***Casp8*** | F: TGCTTGGACTACATCCCACAC | NM_009812* |
| | | R: TGCAGTCTAGGAAGTTGACCA | |
| 28 | ***Bad*** | F: GAGGAGGAGCTTAGCCCTTT | PMID: 32294437# |
| | | R: AGGAACCCTCAAACTCATCG | |
| 29 | ***Myc*** | F: GCTGTTTGAAGGCTGGATTTC | PMID: 32550000# |
| | | R: GATGAAATAGGGCTGTACGGAG | |
| 30 | ***Bax*** | F: TAGCAAACTGGTGCTCAAGG | PMID: 25288756# |
| | | R: TCTTGGATCCAGACAAGCAG | |
| 31 | ***Bcl2l2*** | F: GCGGAGTTCACAGCTCTATAC | NM_007537* |
| | | R: AAAAGGCCCCTACAGTTACCA | |
| 32 | ***Bcl2*** | F: GATGACTGAGTACCTGAACCG | PMID: 25830089# |
| | | R: CAGAGACAGCCAGGAGAAATC | |
| 33 | ***Villin1*** | F: TCAAAGGCTCTCTCAACATCAC | NM_009509* |
| | | R: AGCAGTCACCATCGAAGAAGC | |
| 34 | ***Epcam*** | F: GCGGCTCAGAGAGACTGTG | NM_008532* |
| | | R: CCAAGCATTTAGACGCCAGTTT | |
| 35 | ***Vimentin*** | F: CGTCCACACGCACCTACAG | NM_011701* |
| | | R: GGGGGATGAGGAATAGAGGCT | |
| 36 | ***Lgr5*** | F: CCTACTCGAAGACTTACCCAGT | NM_010195* |
| | | R: GCATTGGGGTGAATGATAGCA | |
| 37 | ***Olfm4*** | F: AAACAATGTCCTTAGCATTCGCC | NM_001030294* |
| | | R: GCTTCCAAGGGCCAATGAAAC | |
| 38 | ***Sox9*** | F: AGTACCCGCATCTGCACAAC | NM_011448* |
| | | R: ACGAAGGGTCTCTTCTCGCT | |
| 39 | ***Sox4*** | F: GACCTGCTCGACCTGAACC | NM_009238* |
| | | R: ACTCCAGCCAATCTCCCGA | |
| 40 | ***Ngn3*** | F: CCAAGAGCGAGTTGGCACT | NM_009719* |
| | | R: CGGGCCATAGAAGCTGTGG | |
| 41 | ***Chga*** | F: ATCCTCTCTATCCTGCGACAC | NM_007693* |
| | | R: GGGCTCTGGTTCTCAAACACT | |
| 42 | ***Cck*** | F: AAGAGCGGCGTATGTCTGTG | NM_031161* |
| | | R: CATCCAGCCCATGTAGTCCC | |

| Sl. No. | Gene | Gene sequence (5`-3`) | GenBank Accession number/Ref. |
|---|---|---|---|
| 43 | ***Ffar2*** | F: CTTGATCCTCACGGCCTACAT | NM_146187* |
| | | R: CCAGGGTCAGATTAAGCAGGAG | |
| 44 | ***Ffar3*** | F: CTAAACCTGACCATTTCGGACC | NM_001033316* |
| | | R: GATAGGCCACGCTCAGAAAAC | |
| 45 | ***Mct2*** | F: GCTGGGTCGTAGTCTGTGC | NM_011391* |
| | | R: ATCCAAGCGATCTGACTGGAG | |
| 46 | ***Mct1*** | F: TGTTAGTCGGAGCCTTCATTTC | NM_009196* |
| | | R: CACTGGTCGTTGCACTGAATA | |
| 47 | ***Glp1r*** | F: ACGGTGTCCCTCTCAGAGAC | NM_021332* |
| | | R: ATCAAAGGTCCGGTTGCAGAA | |
| 48 | ***Neurog2*** | F: AACTCCACGTCCCCATACAG | NM_009718* |
| | | R: GAGGCGCATAACGATGCTTCT | |
| 49 | ***Gcg*** | F: TTACTTTGTGGCTGGATTGCTT | NM_008100* |
| | | R: AGTGGCGTTTGTCTTCATTCA | |
| 50 | ***Pcsk1*** | F: AGTTGGAGGCATAAGAATGCTG | NM_013628* |
| | | R: GCCTTCTGGGCTAGTCTGC | |
| 51 | ***Pcsk2*** | F: AGAGAGACCCCAGGATAAAGATG | NM_008792* |
| | | R: CTTGCCCAGTGTTGAACAGGT | |
| 52 | ***Slc5a1*** | F: AATGCGGCTGACATCTCAGTC | NM_019810* |
| | | R: ACCAAGGCGTTCCATTCAAAG | |
| 53 | ***Bdnf*** | F: TCATACTTCGGTTGCATGAAGG | NM_007540* |
| | | R: AGACCTCTCGAACCTGCCC | |
| 54 | ***Calb*** | F: TCTGGCTTCATTTCGACGCTG | NM_009788* |
| | | R: ACAAAGGATTTCATTTCCGGTGA | |
| 55 | ***Bmi1*** | F: ATCCCCACTTAATGTGTGTCCT | NM_007552* |
| | | R: CTTGCTGGTCTCCAAGTAACG | |
| 56 | ***Chat*** | F: CCATTGTGAAGCGGTTTGGG | NM_009891* |
| | | R: GCCAGGCGGTTGTTTAGATACA | |
| 57 | ***Dbh*** | F: GAGGCGGCTTCCATGTACG | NM_138942* |
| | | R: TCCAGGGGGATGTGGTAGG | |
| 58 | ***Drd3*** | F: CCTCTGAGCCAGATAAGCAGC | NM_007877* |
| | | R: AGACCGTTGCCAAAGATGATG | |
| 59 | ***Elavl3*** | F: ATGGTCACTCAGATACTGGGG | NM_010487* |
| | | R: TTCTGGGGTAGGTAGTTGACG | |
| 60 | ***Fabp7*** | F: GGACACAATGCACATTCAAGAAC | NM_021272* |
| | | R: CCGAACCACAGACTTACAGTTT | |
| 61 | ***Gfap*** | F: GGGGCAAAAGCACCAAAGAAG | NM_001131020* |
| | | R: GGGACAACTTGTATTGTGAGCC | |
| 62 | ***Mef2c*** | F: GTCAGTTGGGAGCTTGCACTA | NM_001170537* |
| | | R: CGGTCTCTAGGAGGAGAAACA | |
| 63 | ***Myogenin*** | F: GAGACATCCCCCTATTTCTACCA | NM_031189* |
| | | R: GCTCAGTCCGCTCATAGCC | |

| Sl. No. | Gene | Gene sequence (5`-3`) | GenBank Accession number/Ref. |
|---|---|---|---|
| 64 | ***Ngfr*** | F: CTAGGGGTGTCCTTTGGAGGT | NM_033217* |
| | | R: CAGGGTTCACACACGGTCT | |
| 65 | ***Nos1*** | F: CTGGTGAAGGAACGGGTCAG | NM_008712* |
| | | R: CCGATCATTGACGGCGAGAAT | |
| 66 | ***Phox2b*** | F: TACGCCGCAGTTCCATACAAACTC | PMID: 30626698# |
| | | R: TCTTTGAGCTGCGCGCTTGTGAAG | |
| 67 | ***Ret*** | F: GCATGTCAGACCCGAACTGG | PMID: 25986924# |
| | | R: CGCTGAGGGTGAAACCATCC | |
| 68 | ***S100b*** | F: TGGTTGCCCTCATTGATGTCT | PMID: 31043739# |
| | | R: CCCATCCCCATCTTCGTCC | |
| 69 | ***Sox10*** | F: GAAGCCCCACATCGACTTCG | NM_011437* |
| | | R: GGCAGGTATTGGTCCAGCTC | |
| 70 | ***Tac1*** | F: ATTCCTTTGTTGGACTAATGGGC | NM_145123* |
| | | R: ACGTCTTCTTTCGTAGTTCTGC | |
| 71 | ***Tert*** | F: GCACTTTGGTTGCCCAATG | NM_009354* |
| | | R: GCACGTTTCTCTCGTTGCG | |
| 72 | ***Htr1a*** | F: GACAGGCGGCAACGATACT | NM_008308* |
| | | R: CCAAGGAGCCGATGAGATAGTT | |
| 73 | ***Tubb3*** | F: TAGACCCCAGCGGCAACTAT | NM_001080971* |
| | | R: GTTCCAGGTTCCAAGTCCACC | |
| 74 | ***Vip*** | F: AGTGTGCTGTTCTCTCAGTCG | NM_011702* |
| | | R: GCCATTTTCTGCTAAGGGATTCT | |
| 75 | ***Uchl1*** | F: AGGGACAGGAAGTTAGCCCTA | NM_011670* |
| | | R: AGCTTCTCCGTTTCAGACAGA | |
| 76 | ***Mcp1*** | F: TTAAAAACCTGGATCGGAACCAA | NM_011333* |
| | | R: GCATTAGCTTCAGATTTACGGGT | |
| 77 | ***18s*** | F: GCAATTATTCCCCATGAACG | PMID: 32302292# |
| | | R: GGCCTCACTAAACCATCCAA | |

* The accession number and primer sequences are optained from Harvard primer bank
# The primer sequence optained from the published article that mentioned herewith PMID

**Supplementary Table S2.** RFA (Randon forest analysis) of intestinal specific marker gene expression in mice administration with Young vs Old FMT (fecal microbiome transplant)

| FMT administration to Mice | | | | |
|---|---|---|---|---|
| **Sl. No.** | **Gene** | **Young FMT** | **Old FMT** | **p-Value Young vs Old FMT** |
| 1 | *Muc2* | 0.950 | 0.074 | 0.000 |
| 2 | *Mct1* | 1.026 | 0.264 | 0.000 |
| 3 | *Myogenin* | 1.015 | 0.118 | 0.000 |
| 4 | *Drd3* | 1.000 | 0.287 | 0.000 |
| 5 | *Math1* | 1.035 | 0.085 | 0.000 |
| 6 | *Gfi* | 0.960 | 0.047 | 0.000 |
| 7 | *Sox9* | 0.988 | 4.215 | 0.000 |
| 8 | *Bcl2* | 1.009 | 0.186 | 0.000 |
| 9 | *Zo1* | 0.991 | 0.110 | 0.001 |
| 10 | *Elavl3* | 0.951 | 0.137 | 0.000 |
| 11 | *Gfap* | 0.977 | 3.117 | 0.000 |
| 12 | *Tnfα* | 0.987 | 9.307 | 0.000 |
| 13 | *Casp8* | 1.000 | 2.101 | 0.010 |
| 14 | *Muc13* | 0.988 | 2.150 | 0.008 |
| 15 | *Bcl2l2* | 0.990 | 0.162 | 0.000 |
| 16 | *Muc6* | 1.007 | 0.126 | 0.000 |
| 17 | *Mef2c* | 0.979 | 4.701 | 0.000 |
| 18 | *Bdnf* | 1.030 | 0.249 | 0.000 |
| 19 | *Ffar2* | 0.943 | 0.060 | 0.000 |
| 20 | *Il10* | 1.015 | 0.246 | 0.013 |
| 21 | *Bad* | 0.995 | 10.926 | 0.001 |
| 22 | *Epcam* | 0.926 | 5.458 | 0.000 |
| 23 | *Villin* | 0.931 | 3.490 | 0.003 |
| 24 | *Hes1* | 0.963 | 3.609 | 0.001 |
| 25 | *Tgfβ1* | 1.031 | 0.228 | 0.000 |
| 26 | *Lyz1* | 1.005 | 5.326 | 0.002 |
| 27 | *Ffar3* | 0.982 | 0.091 | 0.000 |
| 28 | *Calb* | 0.995 | 0.202 | 0.006 |
| 29 | *Il6* | 0.980 | 4.825 | 0.000 |
| 30 | *Tert* | 0.943 | 0.164 | 0.000 |

**Supplementary Table S3.** Fold change and p-Value of metabolite abundance in Young vs Old Donor Mice according to RFA (Randon forest analysis)

| Sl. No. | Metabolite | Young | Old | p-Value |
|---|---|---|---|---|
| | | | | Young vs Old |
| 1 | Butyrate | 1.275 | 0.789 | 0.000 |
| 2 | Taurine | 1.485 | 0.639 | 0.002 |
| 3 | Propionate | 1.136 | 0.861 | 0.003 |
| 4 | Cholate | 0.432 | 1.579 | 0.000 |
| 5 | Acetate | 1.402 | 0.764 | 0.000 |
| 6 | Anserine | 0.681 | 1.331 | 0.000 |
| 7 | Methanol | 0.989 | 0.713 | 0.008 |
| 8 | Valine | 0.711 | 1.291 | 0.000 |
| 9 | 3-Phenylpropionate | 1.415 | 0.806 | 0.065 |
| 10 | Glycine | 1.161 | 0.900 | 0.005 |
| 11 | 3-Hydroxyisobutyrate | 1.053 | 0.966 | 0.322 |
| 12 | Total bile acid & cholesterol | 0.579 | 1.259 | 0.000 |
| 13 | 3-Methyl-2-oxovalerate | 0.796 | 1.132 | 0.002 |
| 14 | Xylose | 1.642 | 0.756 | 0.003 |
| 15 | Choline | 1.070 | 0.925 | 0.045 |
| 16 | Isoleucine | 0.850 | 1.126 | 0.005 |
| 17 | 4-Hydroxyisophenylacetate | 1.172 | 0.957 | 0.000 |
| 18 | Tartrate | 0.868 | 1.100 | 0.050 |
| 19 | Arabinose | 0.929 | 1.030 | 0.181 |
| 20 | Ethanolamine | 0.850 | 1.061 | 0.025 |
| 21 | 2-Hydroxyisobutyrate | 1.087 | 0.891 | 0.023 |
| 22 | Leucine | 0.815 | 1.093 | 0.006 |
| 23 | Pyruvate | 1.148 | 0.929 | 0.035 |
| 24 | Glutamine | 1.104 | 0.956 | 0.037 |
| 25 | Hypoxanthine | 1.145 | 0.873 | 0.034 |
| 26 | Formate | 0.889 | 1.120 | 0.014 |
| 27 | Dimethylamine | 0.973 | 1.059 | 0.319 |
| 28 | Furmate | 1.555 | 0.591 | 0.002 |
| 29 | Ethanol | 0.920 | 1.038 | 0.066 |
| 30 | Alanine | 0.976 | 0.957 | 0.334 |

**Supplementary Table S4.** Details of chemicals and reagents used in this study.

| S. No. | Name | Cat. Number | Vendor |
|---|---|---|---|
| **Chemicals and Reagents** | | | |
| 1 | Ampicillin | A0166 | Sigma |
| 2 | Metronidazole | M1547 | Sigma |
| 3 | Neomycin | N6386 | Sigma |
| 4 | Vancomycin | 195540 | MP Biochemicals |
| 5 | polyethylene glycol | 25322-68-3 | Millipore Sigma |
| 6 | Resazurin | R7017 | Sigma Aldrich |
| 7 | L-cysteine-HCl | 2430 | Calbiochem, EMD Millipore Corp. |
| 8 | Butyrate | B5887 | Sigma |
| 9 | Acetate | S5636 | Sigma |
| 10 | Proionate | P5436 | Sigma |
| 9 | 4-kD-FITC (Fluroescein isothiocyanate)-dextran | FD4 | Sigma Aldrich |
| 10 | LBP ELISA kit | HK205-01 | Hycult Biotech Inc. |
| 11 | sCD14 ELISA kit | MC140 | R&D Systems |
| 12 | IL6 ELISA kit | KMC0061 | Invitrogen |
| 13 | TNFα ELISA kit | BMS607-3 | Invitrogen |
| 14 | Fecal Mucin ELISA kit | FFA-MU-KO1 | Cosmo BioCo. Ltd |
| 15 | Hematoxylin and Eosin | 3530-16 | Ricca |
| 16 | Alcian Blue/PAS staining kit | 395B-1KT | Sigma Aldrich |
| 17 | QiaAmp PowerFecal Pro kit (50) | 2830-50 | Qiagen |
| 18 | Miseq reagent kit v3 | MS-102-3003 | Illumina |
| 19 | Agencourt AMPure XP 60ml kit | A63881 | Beckman Coulter Genomics |
| 20 | 0.45 µm pore nylon membrane sterile filter | 431225 | Corning Incorporated |
| 21 | 0.22 µm nylon membrane sterile syringe filter | 379-2215-OEM | EZFlow, Foxx, Life Sciences |
| 22 | Dulbecco`s phosphate buffered saline (DPBS) | 17-512F | BioWhittaker |
| 23 | Trypsin | 25300-054 | Gibco, Life technologies |
| 24 | Bovine serum albumin (BSA) | BP1600-100 | Fisher Scientific |
| 25 | 70µm cell strainer | 352350 | Falcon, Corning Incorporation |
| 26 | Dulbecco's Modified Eagle Medium: Nutrient Mixture F-12 (DMEM/F12) | 11330-032 | Gibco, Life technologies |
| 27 | IntestiCult Organoid Growth Medium | #06005 | Stemcell |
| 28 | Gentamicin | 15710-064 | Gibco, Life technologies |
| 29 | Matrigel Matrix | 354230 | Corning |
| 30 | CATBP | 5903 | Tocris |
| 31 | Pertussis Toxin | PHZ1174 | Invitrogen |
| 32 | 4.5 g/L D-Glucose with L-Glutamine DMEM | 11995-065 | Gibco |
| 33 | Fetal bovine serum (FBS) | 26140-079 | Gibco |
| 34 | 100 U/mL penicillin, 100 U/mL streptomycin | 15140-122 | Gibco |
| 35 | 0.4 µm pore size of 12-well transwell plates | 3460 | Costar, Corning |
| 36 | Trimethylsilylpropanoic acid (TSP) | AC432120010 | ThermoFisher Scientific |
| 37 | HT-29 cells | HTB-38 | ATCC |
| 38 | CMT-93 | CCL-223 | ATCC |
| 39 | RNeasy Mini Kit | 74106 | Qiagen |
| 40 | High-Capacity cDNA reverse transcription kit | 4368814 | Applied Biosystems |
| 41 | PowerUp SYBR Green master mix | 100029284 | Applied Biosystem |

**Supplementary Table S4.** Details of instruments used in this study.

| Instruments | | | |
|---|---|---|---|
| 1 | Fluorescence 96-well plate reader | | PolarStar Omega, BMG Labtech |
| 2 | AmScope microscope | | Nikon Corporation |
| 3 | MiSeq Sequencer | | Illumina |
| 4 | Qubit-3 Fluorimeter | | Invitrogen |
| 5 | Bruker Ascend 400 MHz high-resolution NMR | | Bruker Biospin |
| 6 | Refrigerated Centrifuge machine | | Eppendroff |
| 7 | EVOM2 Epithelial Voltohmmeter | | WPI |
| 8 | NMR | | Bruker BioSpin |
| 9 | 7500 qRTPCR machine | 4351106 | Applied Biosystem |

**Supplementary Table S5. The human gene primer sequence**

| Sl. No. | Gene | Seuence | Asseccion No. |
|---|---|---|---|
| 1 | h*Ffar2* | F: CCGTGCAGTACAAGCTCTCC | NM_005306* |
| | | R: CTGCTCAGTCGTGTTCAAGTATT | |
| 2 | h*Ffar3* | F: TTCACCACCATCTATCTCACCG | NM_005304* |
| | | R: GGAACTCCAGGTAGCAGGTC | |

* The accession number and primer sequences are optained from Harvard primer bank

**Supplementary Table S6. The buk and but gene primer sequence**

| Sl. No. | Gene | Seuence | Asseccion No. |
|---|---|---|---|
| 1 | *But* | F: GCIGAICATTTCACITGGAAYWSITGGCAYATG | PMID: 30212649 |
| | | R: CCTGCCTTTGCAATRTCIACRAANGC | |
| 2 | *Buk* | 5F1: CCATGCATTAAATCAAAAAGC | PMID: 23836895 |
| | | 5F2: CCATGCGTTAAACCAAAAAGC | |
| | | 6R1: AGTACCTCCACCCATGTG | |
| | | 6R2: AATACCTCCGCCCATATG | |
| | | 6R3: AATACCGCCRCCCATATG | |
| 3 | Total Bacteria | F: GCAGGCCTAACACATGCAAGTC | PMID: 23836895 |
| | | R: CTGCTGCCTCCCGTAGGAGT | |
